# Transient expansion and myofibroblast conversion of adipogenic lineage precursors mediate bone marrow repair after radiation

**DOI:** 10.1172/jci.insight.150323

**Published:** 2022-04-08

**Authors:** Leilei Zhong, Lutian Yao, Nicholas Holdreith, Wei Yu, Tao Gui, Zhen Miao, Yehuda Elkaim, Mingyao Li, Yanqing Gong, Maurizio Pacifici, Amit Maity, Theresa M. Busch, Kyu Sang Joeng, Keith Cengel, Patrick Seale, Wei Tong, Ling Qin

**Affiliations:** 1 Department of Orthopaedic Surgery, Perelman School of Medicine, University of Pennsylvania, Philadelphia, Pennsylvania, USA.; 2 Translational Research Program in Pediatric Orthopaedics, Division of Orthopaedic Surgery, Children’s Hospital of Philadelphia, Philadelphia, Pennsylvania, USA.; 3 Department of Orthopaedics, The First Hospital of China Medical University, Shenyang, China.; 4 Division of Hematology, Children’s Hospital of Philadelphia, Philadelphia, Pennsylvania, USA.; 5 Department of Pediatrics, Perelman School of Medicine at the University of Pennsylvania, Philadelphia, Pennsylvania, USA.; 6 Department of Orthopaedics, Union Hospital, Tongji Medical College, Huazhong University of Science and Technology, Wuhan, China.; 7 Department of Biostatistics, Epidemiology and Informatics,; 8 Division of Translational Medicine and Human Genetics,; 9 Department of Radiation Oncology, and; 10 Department of Cell and Developmental Biology, Perelman School of Medicine, University of Pennsylvania, Philadelphia, Pennsylvania, USA.

**Keywords:** Bone Biology, Vascular Biology, Bioinformatics, Bone marrow, Radiation therapy

## Abstract

Radiation causes a collapse of bone marrow cells and elimination of microvasculature. To understand how bone marrow recovers after radiation, we focused on mesenchymal lineage cells that provide a supportive microenvironment for hematopoiesis and angiogenesis in bone. We recently discovered a nonproliferative subpopulation of marrow adipogenic lineage precursors (MALPs) that express adipogenic markers with no lipid accumulation. Single-cell transcriptomic analysis revealed that MALPs acquire proliferation and myofibroblast features shortly after radiation. Using an adipocyte-specific *Adipoq-Cre*, we validated that MALPs rapidly and transiently expanded at day 3 after radiation, coinciding with marrow vessel dilation and diminished marrow cellularity. Concurrently, MALPs lost most of their cell processes, became more elongated, and highly expressed myofibroblast-related genes. Radiation activated mTOR signaling in MALPs that is essential for their myofibroblast conversion and subsequent bone marrow recovery at day 14. Ablation of MALPs blocked the recovery of bone marrow vasculature and cellularity, including hematopoietic stem and progenitors. Moreover, VEGFa deficiency in MALPs delayed bone marrow recovery after radiation. Taken together, our research demonstrates a critical role of MALPs in mediating bone marrow repair after radiation injury and sheds light on a cellular target for treating marrow suppression after radiotherapy.

## Introduction

Radiation therapy has more than 100 years of history as a cancer treatment. Currently over 50% of patients with breast, prostate, cervical, lung, and head and neck cancers; lymphoma; and soft tissue sarcoma are prescribed radiotherapy in conjunction with surgery and chemotherapy in order to eliminate tumor cells. The effectiveness of this therapy relies on the radiation dosage, which is limited by the radiation tolerance of tumor-adjacent normal tissues, including bone.

Bone marrow is the site for hematopoiesis, and hematopoietic cells account for approximately 98% of bone marrow cells. Exposure to ionizing radiation damages the highly proliferative hematopoietic cells housed in the bone marrow, which may cause marrow suppression (1). Late radiation damage often includes destruction of the primary microenvironment necessary to support hematopoiesis, leading to accumulation of adipose tissue in place of active red marrow. Severe marrow suppression often results in prolonged or permanent decreases in white and red blood cell counts and anemia. This is particularly common for most patients with prostate, testicular, and gynecological cancer receiving radiotherapy to the pelvic area (2–6), because pelvic bones and sacrum contain about 35% active bone marrow in adults. The healthy pelvis, including its marrow, can experience incidental radiation doses on the order of 5 Gy during standard radiotherapy regimens treating pelvic tumors (7). Even such low doses of radiation can trigger hypoplasia or aplasia of the bone marrow and could result in bleeding, pancytopenia, poor wound healing, impaired immunity, and predisposition to infection and sepsis (8, 9). Indeed, relatively low radiation doses to bone marrow can exacerbate lymphopenia and lead to poorer outcomes after chemoradiotherapy for a variety of malignancies (10–13). Moreover, the potential systemic effects of localized marrow irradiation may have even greater clinical significance with the increasing use of immunomodulatory therapies (14). In addition to dosimetric methods of marrow sparing, understanding the mechanisms underlying bone marrow damage and recovery after radiation may lead to novel insights into biological methods to spare marrow toxicity.

Histologically, the first report of radiation damage on bone, termed osteitis, described a reduction in bone marrow vasculature following obliterative endarteritis and periarteritis (15). Early loss of vascularization occurs as the results of swelling and vacuolization of endothelial cells. Such damage is often a dose-limiting factor in radiotherapy treatment planning (16). Rodent models confirm that radiation exposure causes a rapid collapse of bone marrow cells, including hematopoietic stem and progenitor cells (HSPCs) and mature hematopoietic cells, and elimination of microvasculature in bone marrow (17–19).

Apart from hematopoietic and endothelial cells, mesenchymal lineage cells are another major component of bone marrow. Decades of studies have demonstrated that these cells provide a supportive microenvironment for hematopoiesis and angiogenesis (20). Using single-cell RNA-sequencing (scRNA-Seq) technique, we recently elucidated the subpopulations of bone marrow mesenchymal cells and delineated in vivo bilineage differentiation routes from the most primitive progenitors to terminally differentiated bone-forming cells and adipocytes (21). Interestingly, we discovered a potentially novel subpopulation of mesenchymal cells that express adipogenic markers but have no lipid accumulation. Based on their location in the differentiation route, we named them marrow adipogenic lineage precursors (MALPs). Our previous studies demonstrated an important role of MALPs in suppressing bone formation and promoting bone resorption in normal bone metabolism (21, 22). In this study, we applied focal radiation of 5 Gy on mouse femurs to mimic the potential dose clinically experienced by healthy tissue during cancer radiotherapy. This modest dose of radiation allows for deeper interrogation of MALPs in mediating marrow recovery after exposure to radiation. By examining the scRNA-Seq data set of bone marrow mesenchymal lineage cells from irradiated mice followed by validation and mechanistic studies, we uncovered a critical role of MALPs in restoring bone marrow cellularity, including HSPCs, and marrow vasculature after radiation injury.

## Results

### Radiation quickly expands bone marrow mesenchymal lineage cells.

**Figure 1:**
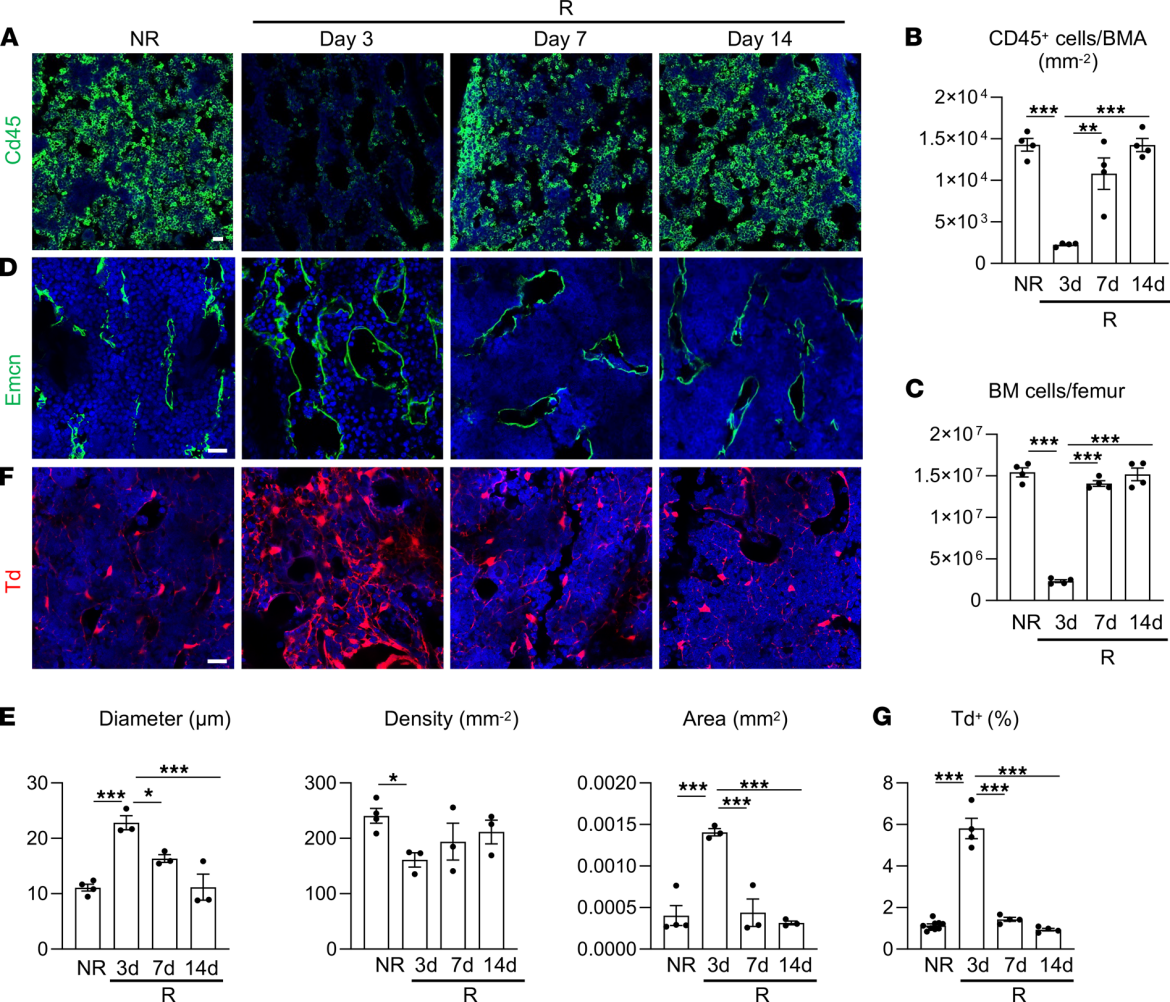
Radiation damage on bone marrow cellularity and vasculature is accompanied by a transient expansion of bone marrow mesenchymal lineage cells. (**A**) Representative fluorescence images of femoral bone marrow CD45^+^ hematopoietic cells after radiation. *Col2/Td* mice received 5 Gy focal radiation at the right femurs. Bones were harvested before radiation (NR) and at days 3, 7, and 14 after radiation (R). Scale bar: 20 μm. (**B**) Quantification of CD45^+^ cells per bone marrow area (BMA). *n* = 4 mice/group. (**C**) Bone marrow (BM) cells were flushed from femurs and counted. *n* = 4 mice/group. (**D**) Representative fluorescence images of bone marrow vasculature stained by Endomucin (Emcn) before and after radiation. Scale bar: 20 μm. (**E**) Quantification of bone marrow vessel diameter, density, and area. *n* = 3–4 mice/group. (**F**) Representative fluorescence images of Td^+^ cells in bone marrow before and after radiation. Scale bar: 20 μm. (**G**) The percentage of Td^+^ cells in bone marrow was quantified by flow cytometry. *n* = 4–7 mice/group. Statistical analysis was performed using 1-way ANOVA with Tukey’s multiple-comparison analysis. *: *P* < 0.05; **: *P* < 0.01; ***: *P* < 0.001.

Radiation causes acute damage on bone marrow cellularity and vasculature. Such damage, if not severe, is often recovered later. We and others showed that *Col2a1-Cre* labels all mesenchymal lineage cells in bone (23, 24). To monitor these cells over time, we applied 5 Gy of focal radiation to the right femurs of 1-month-old *Col2a1-Cre Tomato* (*Col2/Td*) mice at day 0 and found that bone marrow CD45^+^ hematopoietic cells and bone marrow cellularity were drastically reduced by 84% and 85%, respectively, after 3 days (Figure 1, A–C). Starting from day 7, bone marrow cells were mostly restored, reflecting the repair ability of bone marrow after radiation. Vasculature, mainly consisting of capillaries, is ubiquitously distributed in the bone marrow. Radiation damaged bone marrow endothelial cells and remarkably altered marrow vasculature, resulting in vasodilation and a reduction in vessel density at day 3 (Figure 1, D and E). Similar to bone marrow cellularity, vessel damage was mostly repaired by days 7–14.

To our surprise, at day 3 after radiation, we observed a striking increase of bone marrow Td^+^ cells in *Col2/Td* mice, which then returned to normal at days 7–14 (Figure 1F). Flow cytometry confirmed this transient expansion of Td^+^ cells (Figure 1G). Interestingly, the time course of mesenchymal lineage cell expansion and disappearance correlated well with that of bone marrow damage and repair, respectively, promoting us to further investigate their relationship.

### ScRNA-Seq analysis predicts cell cycle entry and myofibroblast conversion of MALPs.

**Figure 2:**
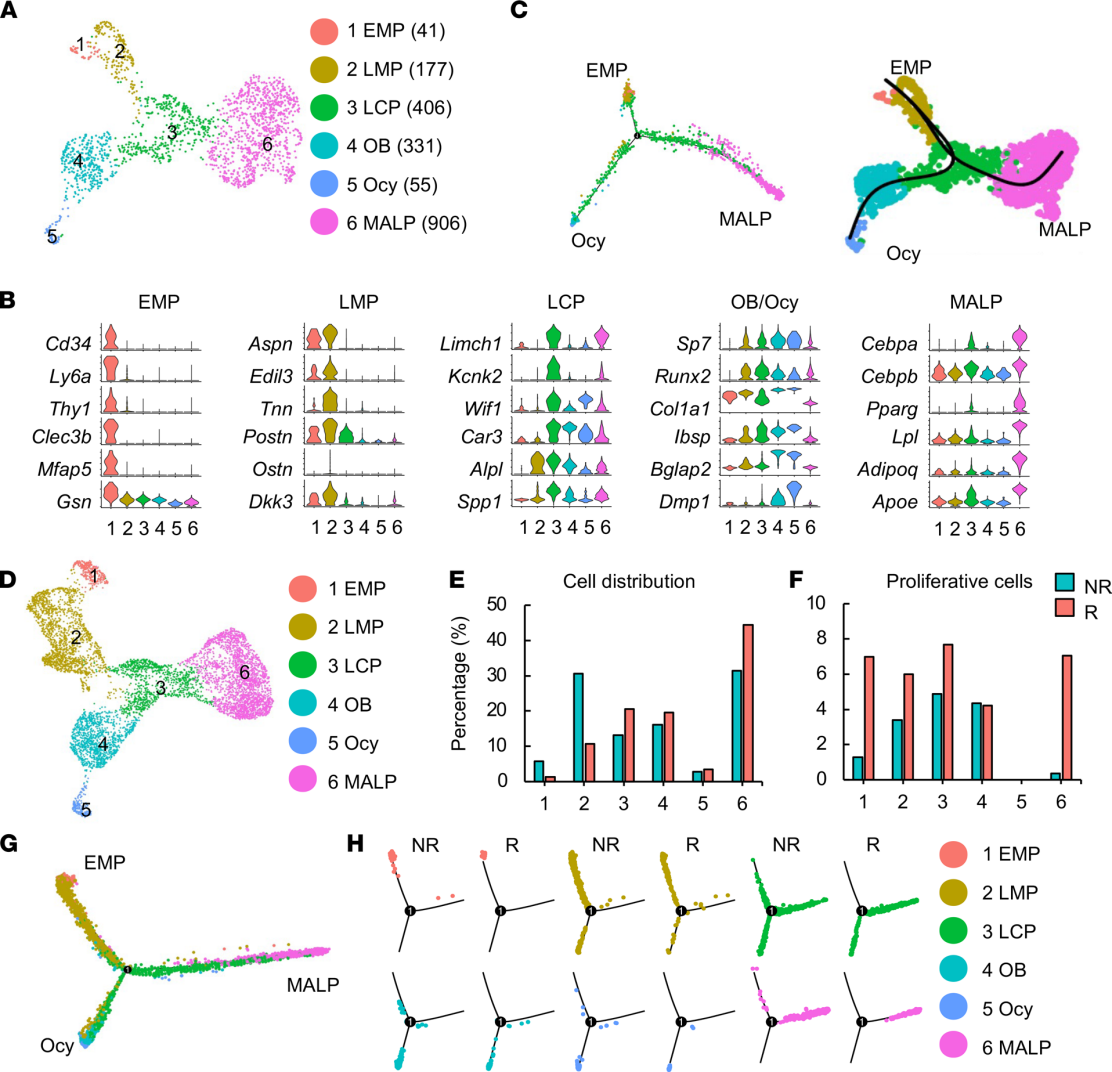
Large-scale scRNA-Seq analysis of Td^+^ cells from 1-month-old *Col2/Td* mouse femurs predicts cell cycle entry of MALPs after radiation. (**A**) The uniform manifold approximation and projection (UMAP) plot of sequenced mesenchymal lineage cells at 3 days after radiation (*n* = 5 mice). Mice received 5 Gy focal radiation to the right femurs. Three days later, right femurs were collected to isolate Td^+^ cells followed by scRNA-Seq. Cell numbers are listed in parentheses next to cluster names. EMP, early mesenchymal progenitor; LMP, late mesenchymal progenitor; LCP, lineage-committed progenitor; OB, osteoblast; Ocy, osteocyte; MALP, marrow adipogenic lineage precursor. (**B**) Violin plots of marker gene expression for indicated cell clusters. (**C**) Monocle (left) and slingshot (right) trajectory plots of sequenced mesenchymal lineage cells. (**D**) An integrated UMAP plot of nonirradiated (NR) and irradiated (R) data sets of mesenchymal lineage cells. (**E**) The percentage of cells in each cluster was calculated in NR and R data sets. (**F**) The percentage of proliferative cells (S/G2/M phase) among each cluster was quantified. (**G**) Monocle trajectory plot of integrated NR and R data sets of mesenchymal lineage cells. (**H**) Monocle trajectory plot of individual cell clusters from integrated NR and R data sets.

Next, we performed large-scale scRNA-Seq on Td^+^ cells sorted from irradiated endosteal bone marrow of 1-month-old *Col2/Td* mice at day 3 after radiation. After quality control, we profiled 2401 cells with an average of 18,801 unique molecular identifiers (UMIs) per cell and an average of 3772 genes per cell (Supplemental Figure 1A; supplemental material available online with this article; https://doi.org/10.1172/jci.insight.150323DS1). Among them, 2294 cells were mesenchymal cells, 19 were hematopoietic cells, 11 were endothelial cells, and 77 were mural cells (Supplemental Figure 1B). Unsupervised clustering of nonchondrogenic mesenchymal cells yielded a similar set of cell clusters (Figure 2A) as we defined previously for endosteal bone marrow mesenchymal lineage cells from healthy 1-month-old mice (Supplemental Figure 2A). According to the expression of cluster-specific markers, mesenchymal subpopulations from both nonirradiated and irradiated mice included EMPs, LMPs, LCPs, osteoblasts, osteocytes, and MALPs (Figure 2B and Supplemental Figure 2B). Specifically, cluster 1 EMPs expressed several common stem cell markers, such as *Ly6a* (*Sca1*), *Cd34*, and *Thy1*; cluster 2 LMPs did not have special markers but strongly expressed *Aspn*, *Edil3*, *Tnn*, *Postn*, and so on compared with cluster 1; cluster 3 LCPs highly expressed *Limch1* and *Kcnk2*; cluster 4 osteoblasts and cluster 5 osteocytes expressed osteogenic markers with gradually increased expression from clusters 1 to 5; and cluster 6 MALPs expressed adipogenic markers at a much higher level than all other clusters. Pseudotime trajectory analyses of the irradiated mouse data set revealed that EMPs, osteocytes, and MALPs were located at 3 ends of the trajectory, indicating that they are either the origin of all cells or terminally differentiated cells (Figure 2C). This result is consistent with pseudotime analysis of healthy bone marrow mesenchymal subpopulations (Supplemental Figure 2C).

Merging irradiated and nonirradiated mouse data sets generated a UMAP plot with the same cell clusters as above (Figure 2D). Hierarchy analysis showed a distinct gene expression signature in each cluster (Supplemental Figure 3). EMPs and LMPs were drastically shrunken in the irradiated sample while LCPs and MALPs were expanded (Figure 2E). Interestingly, cell cycle analysis revealed that the percentages of proliferative cells in EMPs, LMPs, LCPs, and MALPs were all increased in the irradiated data set (Figure 2F). Accordingly, violin plots showed that several cell cycle–promoting genes were upregulated in those clusters within the irradiated data set compared with the healthy data set (Supplemental Figure 4). Interestingly, Adam17 (a disintegrin and a metalloproteinase domain 17), a sheddase for growth factors and cytokines (25), was also stimulated by radiation, which might mediate the proliferation effects of radiation on cells. For this study, we were particularly interested in MALPs because of their nonproliferative nature in nonirradiated mice. Here, our computational analysis predicted cell cycle entry of MALPs after radiation.

Pseudotime trajectory analyses of the merged data set again generated a *Y* shape curve similar to the curve derived from the individual data set (Figure 2G). Separating healthy and irradiated cells based on cell clusters suggested that radiation promoted differentiation. For example, LCPs were evenly distributed among 3 branches (progenitorial, adipogenic, and osteogenic) in the healthy data set but were placed mainly along 2 differentiated branches (adipocyte and osteocyte) in the irradiated data set. MALPs were more shifted toward the terminal end of the adipogenic branch after radiation (Figure 2H).

**Figure 3:**
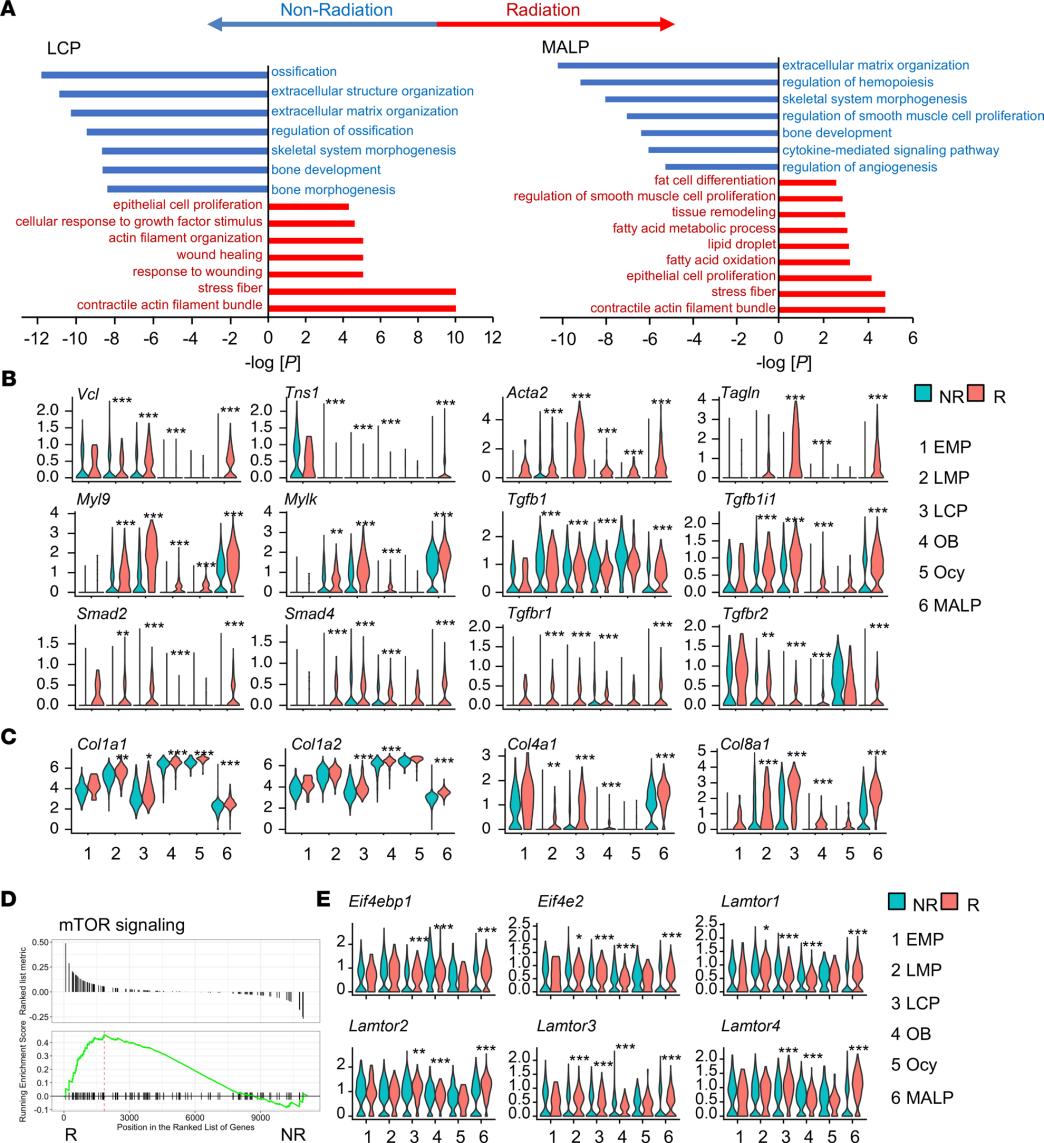
Large-scale scRNA-Seq analysis predicts myofibroblast conversion of LCPs and MALPs after radiation. (**A**) GO term and KEGG pathway analyses of genes differentially regulated in LCPs and MALPs after radiation. (**B**) Violin plots of myofibroblast markers and genes in TGF-β signaling pathway. (**C**) Violin plots of extracellular matrix collagen genes. (**D**) GSEA plot of mTOR signaling pathway of MALPs in NR and R groups. (**E**) Violin plot of mTOR signaling pathway gene markers. Statistical analysis was performed using “bimod” test.use in Findmarkers function. *: *P* < 0.05; **: *P* < 0.01; ***: *P* < 0.001 R vs. NR.

Comparing normal and irradiated cells in each cell cluster of the merged data set generated cluster-specific differentially expressed genes (DEGs). Gene Ontology (GO) term and Kyoto Encyclopedia of Genes and Genomes (KEGG) analyses of DEGs in EMPs, LMPs, LCPs, and MALPs revealed that some common biological activities and pathways, such as extracellular structure organization, wound healing, epithelial cell proliferation, and smooth muscle cell proliferation, were altered after radiation (Figure 3A and Supplemental Figure 5). Meanwhile, radiation had specific effects on individual subpopulations. Pathways such as stress fiber and contractile actin filament bundle, which are related to myofibroblast formation, were particularly enriched in irradiated LCPs and MALPs. In EMPs, radiation downregulated ribosome biogenesis, a pathway vital for stem cell homeostasis (26). In LMPs, bone mineralization and development pathways were downregulated. In osteoblasts and osteocytes, ossification was downregulated and mitotic checkpoint was upregulated. Furthermore, regulon analysis of combined data sets revealed transcription factors whose activity was elevated or suppressed in MALPs after radiation (Supplemental Figure 6). Interestingly, LCPs were the only other cell cluster that shared similar changes in transcription factor activity as MALPs.

Violin plots of myofibroblast-related genes further indicated the myofibroblast conversion of LCPs and MALPs after radiation (Figure 3B). These genes included early myofibroblast markers *Vcl* and *Tns1* (27), mature myofibroblast markers *Acta2* and *Tagln* (28), and actin contractile genes *Myl9* and *Mylk* (29). TGF-β1 is considered a major growth factor promoting myofibroblast development (30). Indeed, *Tgfb1*, its receptors *Tgfbr1* and *Tgfbr2*, and downstream targets *Smad2*, *Smad4*, and *Tgfb1i1* were all upregulated in LCPs and MALPs after radiation (Figure 3B). Myofibroblasts are the primary ECM-secreting cells during wound healing (31). Our scRNA-Seq analysis also revealed that the expression of many ECM collagen genes, such as *Col1a1*, *Col1a2*, *Col4a1*, and *Col8a1*, was highly elevated after radiation (Figure 3C). Pseudotime analysis revealed that under normal conditions, myofibroblast markers, such as *Acta2*, *Tagln*, and *Myl9*, were expressed more in the adipogenic route than in the osteogenic route (Supplemental Figure 7). After radiation, while they were all elevated in both differentiation routes, the enhancement was more drastic in the adipogenic route. Gene set enrichment analysis (GSEA) revealed that the mTOR signaling pathway, another pathway promoting myofibroblast formation (32, 33), was elevated in MALPs after radiation (normalized enrichment score = 1.32, *P* = 0.034, Figure 3D). Violin plots show that radiation increased the expression of several genes related with the mTOR pathway in MALPs (Figure 3E). In summary, scRNA-Seq analyses predict that mesenchymal lineage cells, especially LCPs and MALPs, acquire a myofibroblastic phenotype after radiation.

### Radiation transiently expands MALP pool via stimulating its proliferation.

**Figure 4:**
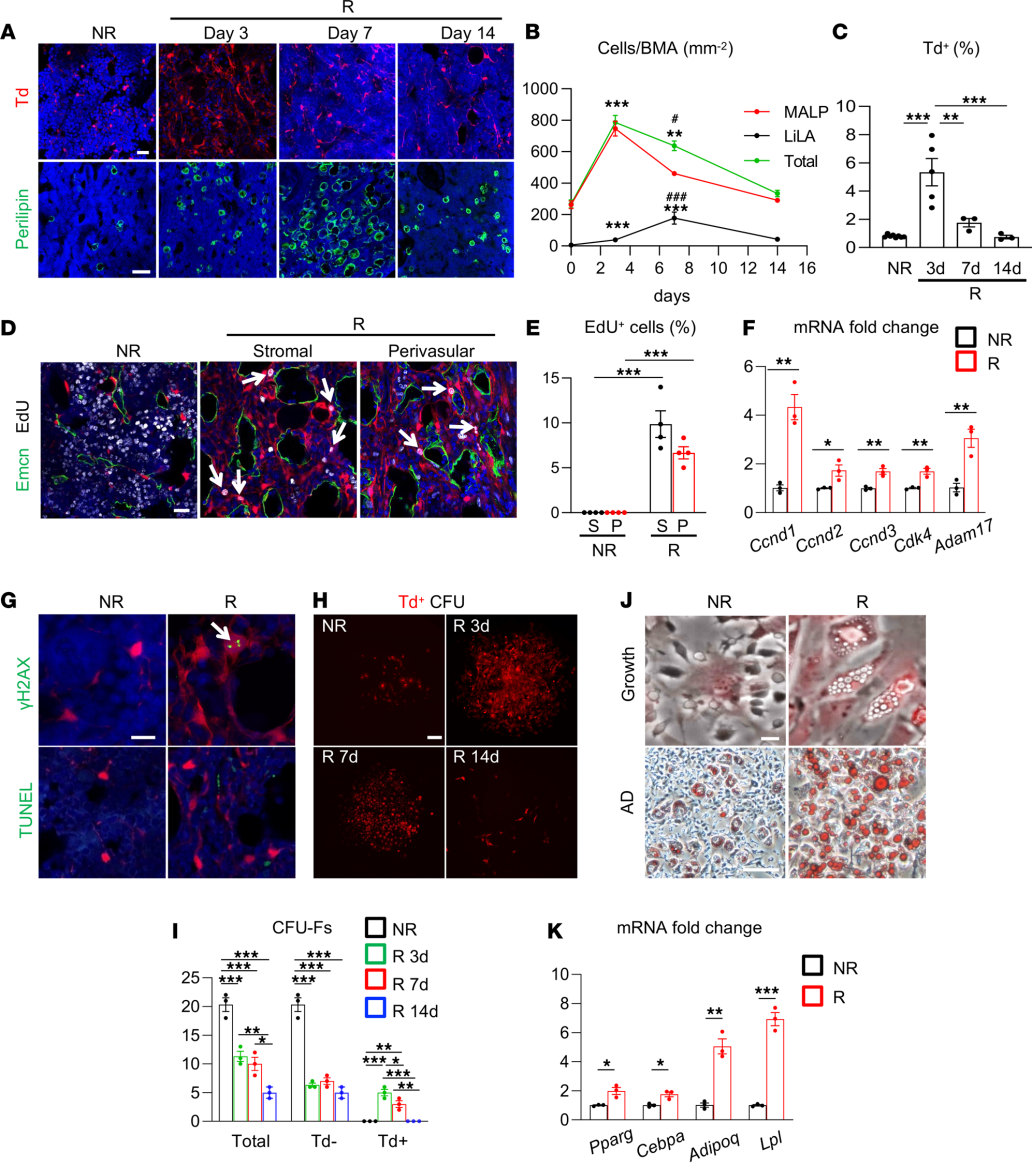
Radiation quickly promotes MALP expansion. (**A**) Representative fluorescence images of Td^+^ cells (top) and Perilipin^+^ lipid-laden adipocytes (LiLAs) (bottom) in the bone marrow of 1-month-old *Adipoq/Td* femur before (NR) and after (R) focal radiation. Scale bar: 20 μm (top) and 100 μm (bottom). (**B**) The time course change of MALPs, LiLAs, and total adipogenic lineage cells (MALPs plus LiLAs) after radiation. BMA, bone marrow area. *n* = 3–5 mice/group. (**C**) Flow analysis of Td^+^ cells in bone marrow. *n* = 3–5 mice/group. (**D**) Representative fluorescence images of EdU incorporation in stromal and perivascular cells of *Adipoq/Td* mice before and at 3 days after radiation. Arrows point to EdU^+^Td^+^ cells. Scale bar: 20 μm. (**E**) Quantification of EdU^+^ cells in Td^+^ stromal cells (S) and pericytes (P). *n* = 4 mice/group. (**F**) qRT-PCR analysis of cell cycle–promoting genes in sorted Td^+^ cells before and at 3 days after radiation. *n* = 3 mice/group. (**G**) Representative fluorescence images of γH2AX and TUNEL staining in bone marrow. An arrow points to γH2AX foci. Scale bar: 10 μm. (**H**) Representative fluorescence images of CFU-F colonies from *Adipoq/Td* bone marrow with or without focal radiation. Scale bar: 100 μm. (**I**) Quantification of Td^–^, Td^+^, and total CFU-F colonies per 1 million bone marrow cells before and after radiation. *n* = 3 mice/group. (**J**) Representative Oil Red O staining of mesenchymal progenitors from nonirradiated (NR) and irradiated femoral bone marrow cultured in growth (top) and adipogenic (AD, bottom) medium. Scale bar: 20 μm (top) and 100 μm (bottom). (**K**) qRT-PCR analysis of adipogenic markers in mesenchymal progenitors cultured in adipogenic medium. *n* = 3 mice/group. Statistical analysis was performed using 1-way ANOVA with Tukey’s multiple-comparison analysis (**B**, **C**, **E**, and **I**) or nonparametric Student’s *t* test (**F** and **K**). *: *P* < 0.05; **: *P* < 0.01; ***: *P* < 0.001 (day 3 or day 7 vs. day 0). ^#^: *P* < 0.05; ^###^: *P* < 0.001. (day 7 vs. day 14).

We previously demonstrated that *Adipoq-Cre* labels MALPs in mouse bone marrow (21, 22). To validate the above computational predictions, we subjected 1-month-old *Adipoq-Cre Tomato* (*Adipoq/Td*) mice to focal radiation on their right femurs. Similar to *Col2/Td* mice, *Adipoq/Td* mice displayed a remarkable increase of Td^+^ cells at day 3 after radiation, then a gradual decline at later time points (Figure 4, A and B). These changes were validated by flow cytometry (Figure 4C). In line with the notion that radiation increases bone marrow adiposity, LiLAs, which are Perilipin^+^, peaked at day 7. These changes occurred similarly in both metaphyseal and diaphyseal bone regions.

To investigate whether increased MALPs are the source for LiLAs after radiation, we performed a lineage tracing experiment using *Adipoq-CreER Td* (*AdipoqER/Td*) mice. These mice received tamoxifen at P14–P16, when LiLAs are absent, and focal radiation at their right femurs at P17. Almost all Perilipin^+^ LiLAs at 3, 7, and 14 days later were Td^+^ (Supplemental Figure 8A), suggesting that MALPs become LiLAs. In both *Adipoq/Td* and *AdipoqER/Td* mice, MALPs, LiLAs, and total adipogenic lineage cells (MALPs plus LiLAs) peaked at days 3, 7, and 3, respectively, after radiation (Figure 4B and Supplemental Figure 8B).

MALPs exist both as bone marrow stromal cells and as capillary pericytes (21). To detect their proliferative status, 1-month-old *Adipoq/Td* mice were subjected to focal radiation and an EdU injection before tissue harvest. In healthy bone marrow, Td^+^ cells, regardless of its location, did not have any EdU incorporation (Figure 4, D and E). On the contrary, after radiation, while EdU signal was largely diminished inside the bone marrow, a substantial portion of Td^+^ stromal cells (9.9%) and pericytes (6.7%) became EdU^+^, indicating that radiation stimulated their proliferation. Moreover, quantitative real-time PCR (qRT-PCR) assay of sorted Td^+^ cells from *Adipoq/Td* mice with or without radiation confirmed that cell cycle–promoting genes predicted by scRNA-Seq analysis were upregulated after radiation (Figure 4F). Radiation did induce DNA DSBs but not apoptosis in MALPs, as shown by γH2AX and TUNEL staining, respectively (Figure 4G).

A colony-forming unit fibroblast (CFU-F) assay detects proliferative mesenchymal lineage cells. Under normal circumstances, bone marrow cells from 1-month-old *Adipoq/Td* mice only formed Td^–^ CFU-F colonies, indicating that MALPs are not proliferative. Strikingly, while radiation drastically reduced total and Td^–^ CFU-F number, it promoted the formation of Td^+^ CFU-F colonies (Figure 4, H and I). In the nonirradiated group, we observed that some Td^+^ cells did attach to the dish but did not grow into a full colony. On the contrary, in the radiated groups, many CFU-F colonies were made of 100% Td^+^ cells. They peaked at day 3 and declined afterward. Interestingly, Td^+^ cells from irradiated mice, but not from normal mice, developed spontaneous adipogenesis even in the growth medium (Figure 4J). When cultured in adipogenic differentiation medium for 5 days, bone marrow mesenchymal cells from radiated mice contained many more lipid droplets (Figure 4J) and expressed many more adipogenic markers than those cells from nonradiated mice (Figure 4K). Note that all lipid-containing cells were Td^+^ in both groups. Taken together, the above assays confirmed that MALPs, which are nonproliferative cells, acquire proliferation ability quickly after radiation.

### Radiation converts MALPs into myofibroblasts.

**Figure 5:**
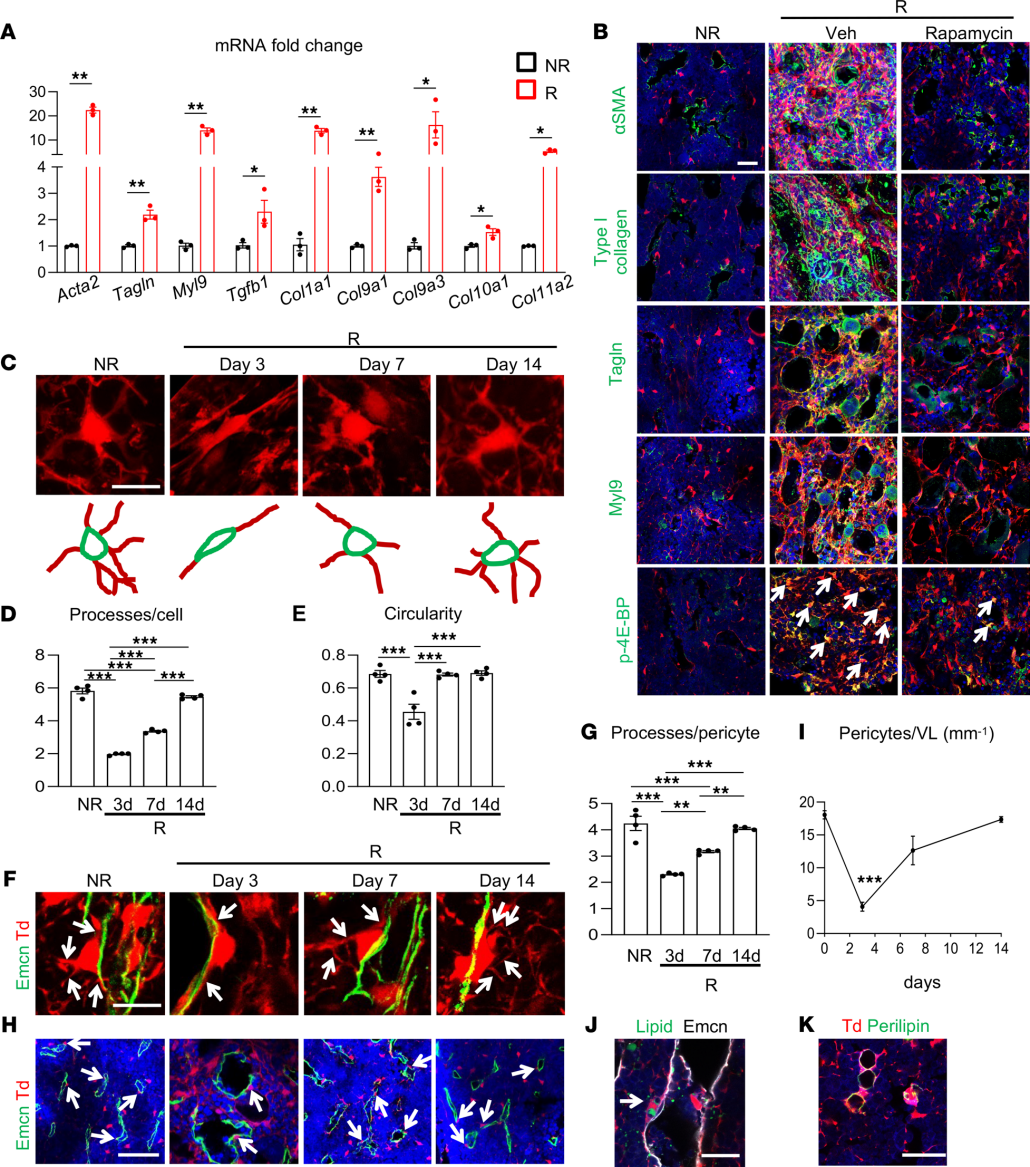
Radiation converts MALPs into myofibroblasts with reduced adherence to vessels. (**A**) The expression of myofibroblast markers was measured by qRT-PCR in sorted bone marrow Td^+^ cells from *Adipoq/Td* mice before (NR) and at day 3 after (R) radiation. *n* = 3 mice/group. (**B**) Immunofluorescence staining of myofibroblast markers (α-SMA, type I collagen, Tagln, and Myl9) and mTOR pathway reporter (phospho–4E-BP, p-4E-BP) in the bone marrow of *Adipoq/Td* mice at day 3 after radiation. Arrows point to p–4E-BP^+^Td^+^ cells. Scale bar: 10 μm. (**C**) Representative 3D images showing cell processes of MALPs before and after radiation (top) and their corresponding cartoons (bottom). Scale bar: 10 μm. (**D**) Quantification of cell processes per Td^+^ cell. *n* = 4 mice/group. (**E**) Quantification of circularity of Td^+^ cells. *n* = 4 mice/group. (**F**) Representative fluorescence images of *Adipoq/Td* femoral bone marrow with Emcn staining (vessels) at a high magnification. Arrows point to cell processes derived from pericytes. Scale bar: 10 μm. (**G**) Quantification of cell processes per pericyte. *n* = 4 mice/group. (**H**) Representative fluorescence images of *Adipoq/Td* femoral bone marrow with Emcn staining (vessels) at a low magnification. Arrows point to Td^+^ pericytes. Scale bar: 20 μm. (**I**) Td^+^ pericytes were counted in bone marrow over the time after radiation. *n* = 5 mice/group. VL, vessel length. (**J**) A fluorescence image with BODIPY (lipid) and Emcn (vessel) staining shows a Td^+^ pericyte with lipid accumulation (indicated by an arrow). Scale bar: 30 μm. (**K**) Perilipin^+^ LiLAs in bone marrow do not have cell processes. Scale bar: 30 μm. Statistical analysis was performed using nonparametric Student’s *t* test (**A**); 1-way ANOVA with Tukey’s multiple-comparison analysis was used (**D**, **E**, **G**, and **I**). *: *P* < 0.05; **: *P* < 0.01; ***: *P* < 0.001 (day 3 vs. day 0 in **I**).

We next characterized the myofibroblastic features of MALPs after radiation as predicted by computational assay. qRT-PCR of sorted bone marrow Td^+^ cells from *Adipoq/Td* mice confirmed that myofibroblast markers, such as *Acta2*, *Tagln*, and *Myl9*, as well as ECM proteins, such as *Col1a1*, *Col91a1*, and *Col11a2*, were all increased at day 3 after radiation (Figure 5A). Immunostaining further validated that α–smooth muscle actin (α-SMA; Acta2), Tagln, Myl9, and type I collagen were substantially increased at the protein level in bone marrow after radiation (Figure 5B). The amount of p–4E-BP1, an indicator of mTOR pathway activity, was also increased in Td^+^ cells. Interestingly, treatment of mice with rapamycin, an mTOR inhibitor, right after radiation, not only diminished p–4E-BP1 amount but also suppressed the increase of myofibroblast markers, suggesting that radiation-induced mTOR pathway activation mediates the myofibroblast conversion of MALPs.

Our previous study demonstrated that MALPs possess a unique shape containing a central cell body and multiple cell processes forming a 3D network structure throughout the bone marrow (21). Strikingly, radiation greatly reduced cell processes and circularity, resulting in a myofibroblastic shape at day 3 (Figure 5, C–E). These shape changes gradually recovered over time.

Confocal 3D imaging showed that bone marrow capillary vessels were wrapped by cell processes extending from both Td^+^ pericytes and Td^+^ stromal cells (21). In line with the overall reduction of cell processes from MALPs, cell processes wrapping vessels were also greatly reduced shortly after radiation (Figure 5, F and G). Interestingly, this was accompanied by a 78% decrease of Td^+^ pericytes covering the vessels (Figure 5, H and I) and an accumulation of lipids in Td^+^ pericytes (Figure 5J). In healthy mice, no pericyte was observed to have lipid. It appears that radiation rapidly accelerated the differentiation of Td^+^ MALPs into LiLAs, which is consistent with the GO term analysis finding that radiation upregulated pathways related to fat cell differentiation, fatty acid metabolic process, lipid droplet, and fatty acid oxidation in MALPs (Figure 3A). Note that LiLAs did not possess cell processes (Figure 5K). Thus, the loss of cell processes likely causes the detachment of pericytes from vessels, explaining the paradox that radiation increases total MALPs but decreases the pericyte portion of MALPs.

### MALPs are indispensable for bone marrow repair after radiation.

**Figure 6:**
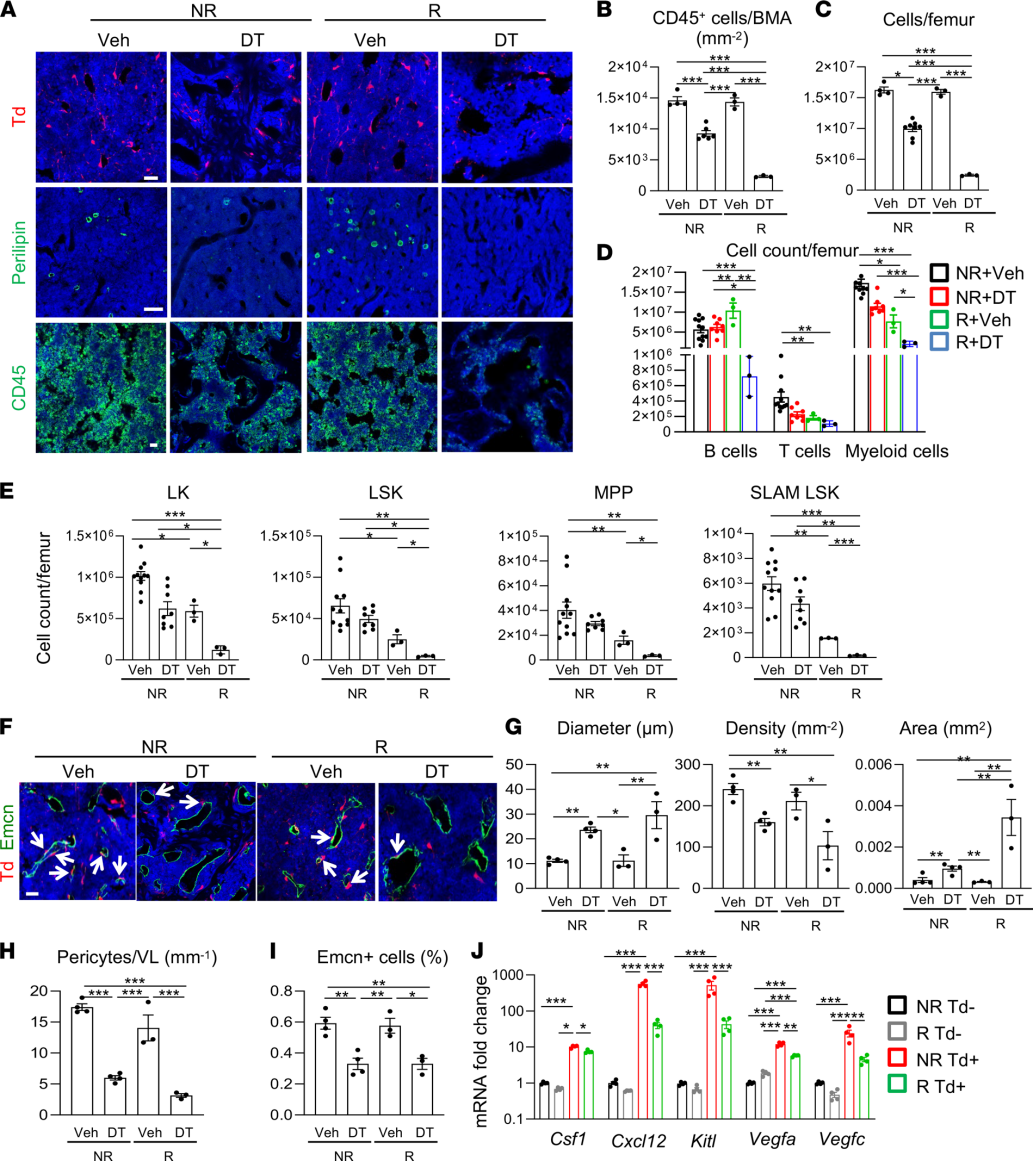
MALP ablation blocks bone marrow recovery after radiation. (**A**) Representative fluorescence images of Td^+^ cells, Perilipin^+^ LiLAs, and CD45^+^ hematopoietic cells in femoral bone marrow of *Adipoq/Td/DTR* mice after receiving 2 weeks of vehicle (Veh) or DT injections with or without prior radiation. Scale bar: 20 μm (top), 100 μm (middle), and 20 μm (bottom). (**B**) Quantification of CD45^+^ cells per bone marrow area. *n* = 3–6 mice/group. (**C**) Bone marrow cells were flushed from femurs and counted. *n* = 3–8 mice/group. (**D**) Cell counts of hematopoietic lineage cells in the bone marrow. *n* = 3–11 mice/group. B cells = B220^+^, T cells = CD3^+^, myeloid cells = Gr1^+^ and/or Mac1^+^. (**E**) Cell counts of HSPCs. *n* = 3–11 mice/group. LK, Lineage^–^cKit^+^, LSK, Lineage^–^Sca1^+^cKit^+^, SLAM LSK, Lineage^–^Sca1^+^cKit^+^CD48^–^CD150^+^, MPP, Lineage^–^Sca1^+^cKit^+^CD48^+^CD150^–^. (**F**) Representative fluorescence images of *Adipoq/Td/DTR* femoral bone marrow with Emcn staining (vessels). Arrows point to Td^+^ pericytes. Scale bar: 20 μm. (**G**) Quantification of bone marrow vessel diameter, density, and area. (**H**) The number of pericytes per vessel length (VL) was measured. *n* = 3–4 mice/group. (**I**) The percentage of Emcn^+^ endothelial cells in bone marrow was measured by flow cytometry. *n* = 3–4 mice/group. (**J**) qRT-PCR analysis of hematopoietic and angiogenic factors in sorted Td^–^ and Td^+^ cells from bone marrow before and after radiation. *n* = 4 mice/group. Statistical analysis was performed using 1-way ANOVA with Tukey’s multiple-comparison analysis. *: *P* < 0.05; **: *P* < 0.01; ***: *P* < 0.001.

To investigate the role of MALPs in restoring the bone marrow compartment after radiation, we tested the consequences of their ablation using *Adipoq-Cre Rosa-Tomato DTR* (*Adipoq/Td/DTR*) mice. Notably, Td signal did not overlap with either Cd45 or Emcn staining, indicating that hematopoietic cells and endothelial cells were not ablated by this approach (Supplemental Figure 9). Compared with vehicle injections, diphtheria toxin (DT) injections into 1-month-old *Adipoq/Td/DTR* mice for 14 days effectively eliminated Td^+^ cells, including MALPs and Perilipin^+^ LiLAs in bone marrow with or without radiation (Figure 6A). While at this time point bone marrow cellularity was mostly recovered in vehicle-injected mice after radiation, it remained at a very low level in DT-injected mice. Specifically, CD45^+^ hematopoietic cells (Figure 6B) and total bone marrow cells (Figure 6C) were reduced by 81% and 85%, respectively, in the DT group compared with the vehicle group. DT injections in nonirradiated mice also reduced bone marrow cellularity by 39%, partially due to the thickening of metaphyseal trabecular bone and de novo trabecular bone formation (bone volume fraction: ~10.5%) in the diaphyseal bone marrow (Supplemental Figure 10), which is consistent with our previous study (21). However, the decrease of bone marrow cellularity was much more severe under the radiation condition.

Analysis of hematopoietic cells revealed that only B cells, but not T cells, myeloid cells, or HSPCs, were recovered at day 14 after radiation (Figure 6, D and E). DT injections further drastically reduced the number of those cells in bone, suggesting that MALPs are critical for hematopoiesis recovery postradiation. Interestingly, based on our scRNA-Seq data, several cytokines essential for hematopoiesis, such as Cxcl12, IL-7, and Kitl, were highly and specifically expressed in MALPs in both normal and irradiated groups (Supplemental Figure 11). In line with these data, we also observed pairs of Cd150^+^ HSPCs and MALPs in the bone marrow (Supplemental Figure 12), suggesting that MALPs provide niche to HSPCs.

To determine whether disruption of hematopoiesis in the femurs affects overall hematopoiesis, we analyzed peripheral blood (PB) components. To our surprise, white blood cells were greatly reduced in the PB of both vehicle-treated mice and DT-injected mice at day 14 after radiation (Supplemental Figure 13), indicating that the contribution of HSPCs within the femurs to the whole-body hematopoiesis is significant. Interestingly, compared with vehicle injections, DT injections further reduced white blood cells, lymphocytes, and monocytes, but not platelets and erythrocytes, suggesting that continuous disruption of hematopoiesis in the femurs significantly affects overall hematopoiesis.

In addition to hematopoietic cells, bone marrow is highly vascularized. Whereas blood vessels in bone were mostly recovered at day 14 after radiation in vehicle-injected mice, they remained damaged in DT-injected mice (Figure 6F). Specifically, vessel diameter and area increased by 2.6- and 10.9-fold, respectively, while vessel density decreased by 51% (Figure 6G). Meanwhile and as expected, Td^+^ pericytes remained at a very low level in DT-injected mice (Figure 6H), and the number of endothelial cells was significantly decreased as well (Figure 6I). Note that vessels were similarly damaged in DT-treated nonirradiated mice, which is consistent with our previous report (21). These data suggest that MALPs are critical for vessel regeneration in bone marrow. In line with these observations, our scRNA-Seq data sets pointed out that MALPs expressed many angiogenic factors at a much higher level than other mesenchymal subpopulations in both normal and irradiated bone (Supplemental Figure 14), which was further validated by quantitative PCR (Figure 6J). Taken together, our data demonstrate that rapid expansion of MALPs after radiation plays an essential role in the restoration of bone marrow hematopoiesis and the restabilization of marrow vasculature after radiation damage.

Our data indicated that the mTOR pathway mediates the myofibroblast conversion of MALPs. To investigate whether this cell change contributed to the bone marrow recovery, we treated mice with rapamycin after radiation for 14 days. Interestingly, when bone marrow in vehicle-treated mice was recovered at this time point, bone marrow hematopoietic Cd45^+^ cells (Supplemental Figure 15, A and B) and vasculature (Supplemental Figure 15, A and C) were still damaged in the rapamycin group. However, rapamycin did not alter LiLA production (Supplemental Figure 15, D and E). These results suggest that myofibroblast conversion of MALPs is required for repairing bone marrow but not involved in radiation-induced marrow adiposity.

### MALP-derived VEGFa partially mediates bone marrow recovery.

**Figure 7:**
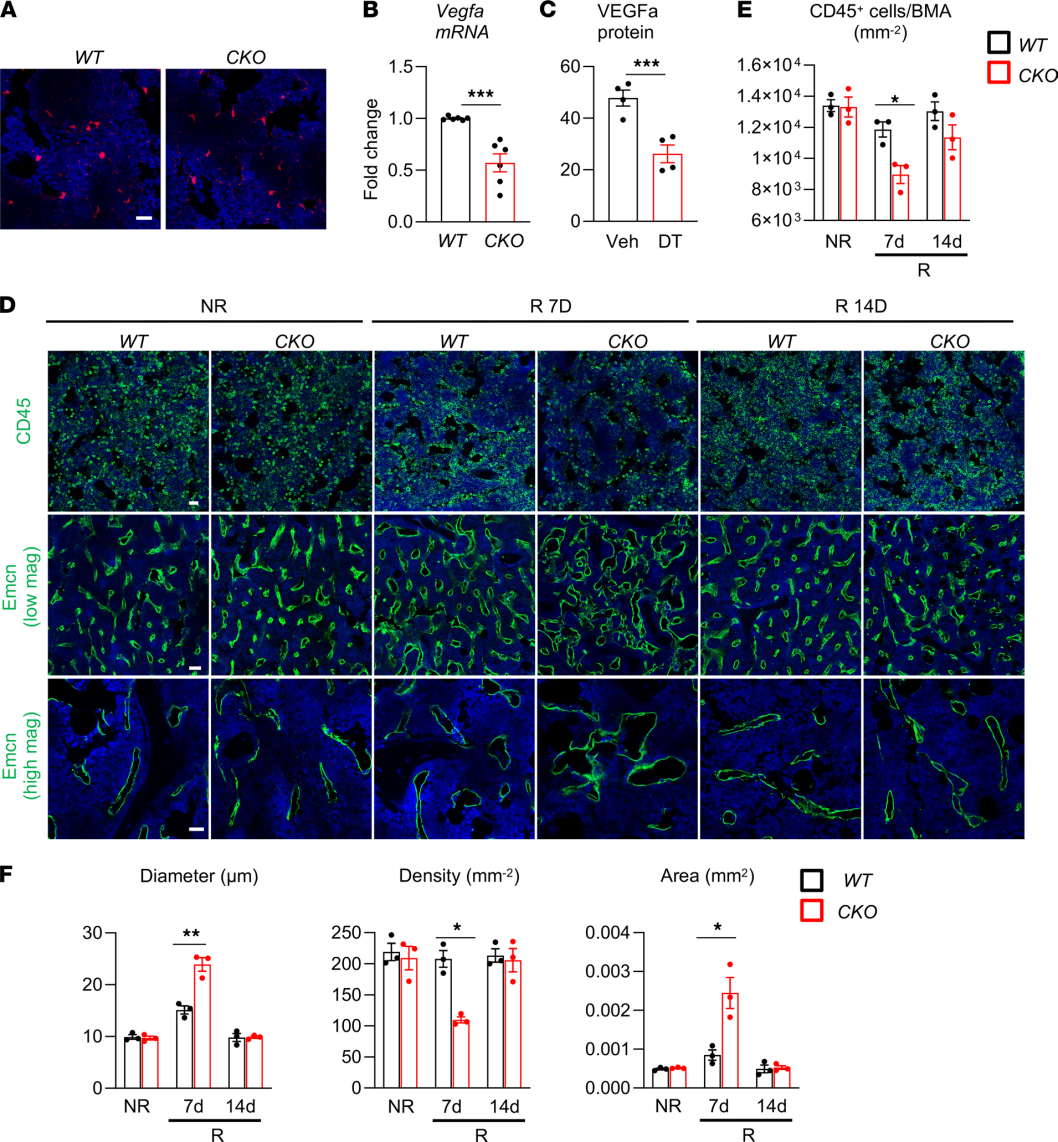
MALP-derived VEGFa partially mediates bone marrow recovery after radiation. (**A**) Representative fluorescence images of bone marrow from *Vegfa-CKO* mice containing Td reporter. Scale bar: 20 μm. (**B**) qRT-PCR analysis of *Vegfa* mRNA in bone marrow of *WT* and *Vegfa-CKO* mice at 2-month-old age. *n* = 6 mice/group. (**C**) Cytokine array reveals that bone marrow VEGFa amount is decreased in *Adipoq/Td/DTR* mice receiving DT injections for 2 weeks. *n* = 4 mice/group. (**D**) Representative CD45 and Emcn fluorescence staining of *WT* and *Vegfa-CKO* femoral bone marrow before (NR) and at days 7 and 14 (R) after radiation. Emcn-stained vessel images are shown at low and high magnifications. Scale bar: 20 μm (top), 50 μm (middle), and 20 μm (bottom). (**E**) CD45^+^ hematopoietic cells were quantified in bone marrow. BMA, bone marrow area. *n* = 3 mice/group. (**F**) Vessel diameter, density, and area were quantified in bone marrow. *n* = 3 mice/group. Statistical analysis was performed using nonparametric Student’s *t* test (**B** and **C**); 2-way ANOVA was used (**E** and **F**). *: *P* < 0.05; **: *P* < 0.01; ***: *P* < 0.001.

Among all the secreted factors highly expressed in MALPs, VEGFa is unique because it is involved in both hematopoiesis and angiogenesis (34). To investigate its role in bone marrow recovery, we constructed *Adipoq-Cre Td Vegfa^fl/fl^* (*Vegfa-CKO*) mice. At 2 months of age, *Vegfa-CKO* mice had the same number of MALPs as WT mice (Figure 7A), but their bone marrow *Vegfa* expression was reduced by 43% (Figure 7B), showing that MALPs are a major source of VEGFa in bone. Additional support for this conclusion came from cytokine array analysis of bone marrow of *Adipoq/Td/DTR* mice. Two weeks of DT injections eliminated MALPs and reduced bone marrow VEGFa level by 45% (Figure 7C).

Without radiation, *Vegfa-CKO* mice had normal bone marrow cellularity and vasculature (Figure 7, D–F), but interestingly, bone marrow recovery was significantly delayed in these mice after radiation. At day 7 after radiation, bone marrow cellularity and vessels appeared mostly recovered in *WT* bones but not in *CKO* bones. Specifically, CD45^+^ bone marrow cells were significantly lower in *CKO* than in *WT* (Figure 7, D and E), and vessels were much more dilated and reduced in number in *CKO* than in *WT* (Figure 7, D and F). By day 14 after radiation, bone marrow had similarly recovered in *WT* and *CKO* mice (Figure 7, D–F). These data demonstrate that VEGFa partially mediates the repair capability of MALPs after radiation injury.

### Aging expands MALPs without converting them into myofibroblasts.

**Figure 8:**
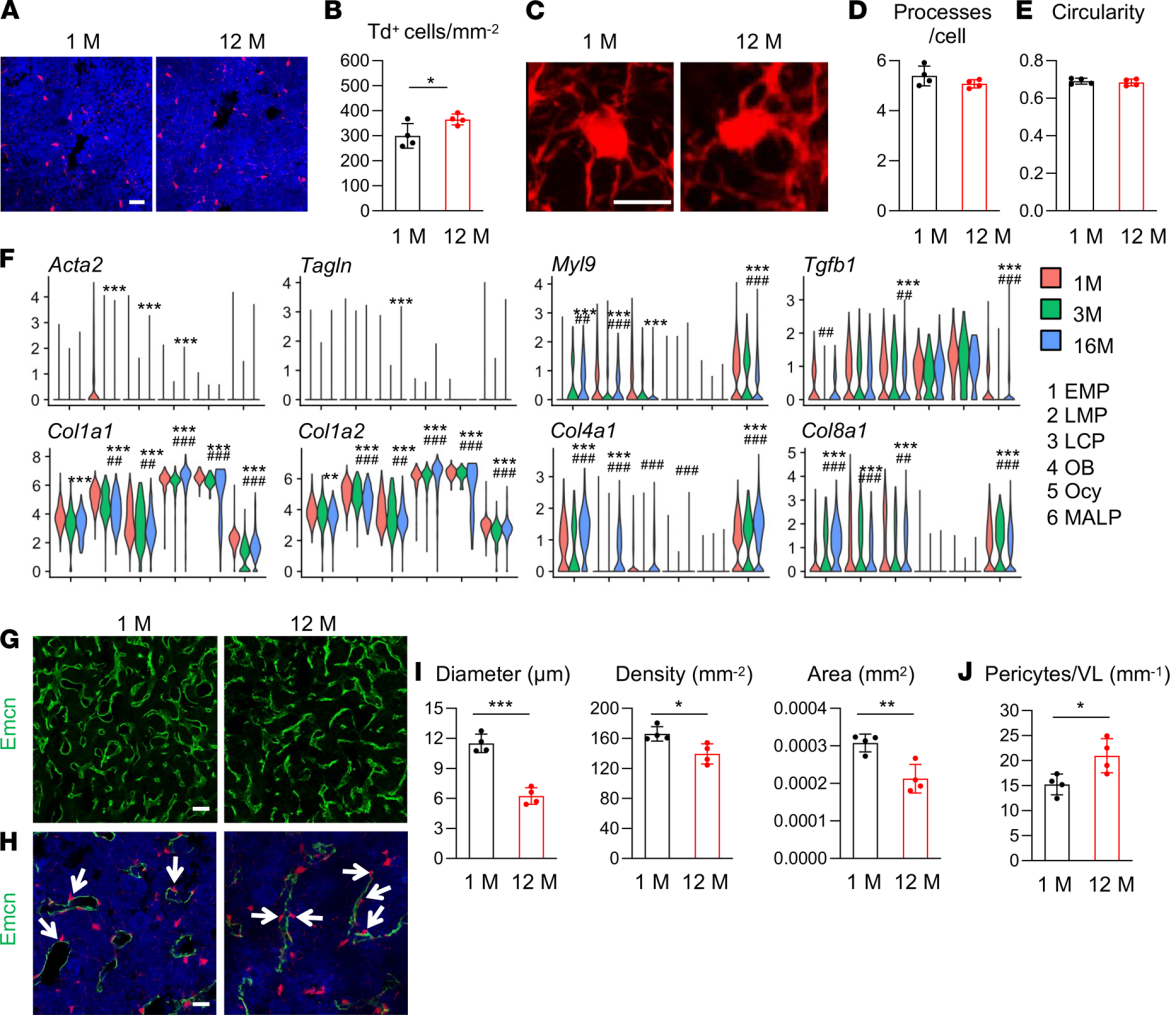
Aging expands MALPs but does not convert them into myofibroblasts. (**A**) Representative fluorescence images of Td^+^ cells in femoral bone marrow of *Adipoq/Td* mice at 1 and 12 months of age. Scale bar: 20 μm. (**B**) Quantification reveals an increase of Td*^+^* cells in bone marrow from aging mice. *n* = 4 mice/group. (**C**) Representative 3D fluorescence images of Td^+^ cells with processes in the bone marrow. Scale bar: 10 μm. (**D**) The number of cell processes in Td^+^ cells was quantified. *n* = 4 mice/group. (**E**) Cell circularity was measured. *n* = 4 mice/group. (**F**) Violin plots show the expression patterns of myofibroblast markers and collagen genes in scRNA-Seq data sets of bone marrow mesenchymal lineage cells from mice at 1, 3, and 16 months of age. (**G**) Fluorescence images of vessel staining in femur at a low magnification. Scale bar: 50 μm. (**H**) Fluorescence images of vessel staining in femur at a high magnification to show Td^+^ pericytes (arrows). Scale bar: 20 μm. (**I**) Vessel diameter, density, and area are quantified in the central bone marrow. *n* = 4 mice/group. (**J**) The number of Td^+^ pericytes was quantified. Statistical analysis was performed using nonparametric Student’s *t* test (**B**, **D**, **E**, **I**, and **J**), *: *P* < 0.05; **: *P* < 0.01; ***: *P* < 0.001; “bimod” test.use in Findmarkers function (**F**). *: *P* < 0.05; **: *P* < 0.01; ***: *P* < 0.001 (16M vs. 1M). ^##^: *P* < 0.01; ^###^: *P* < 0.001 (16M vs. 3M).

Radiation and aging, 2 pathologic events in bone, share some common characteristics, such as increased ROS levels, DNA damage, senescence, and marrow adiposity (35). To understand whether MALPs undergo similar changes during aging, we analyzed Td^+^ cells in the bone marrow of 12-month-old *Adipoq/Td* mice. Compared with those in 1-month-old mice, fluorescence imaging revealed a 22% increase of Td^+^ cells (Figure 8, A and B). Surprisingly, their cell shape and process number remained the same as those in young mice (Figure 8, C–E), suggesting that they did not undergo myofibroblast conversion during aging. We previously performed scRNA-Seq of bone marrow mesenchymal lineage mice at 1, 3, and 16 months of age (21). Analyzing these data sets also did not reveal any increase in the expression of myofibroblast-associated genes during aging (Figure 8F).

Aging also affects marrow vasculature differently from radiation. Instead of dilating, vessels became narrower in aged bone marrow, resulting in decreased vessel diameter, density, and area (Figure 8, G–I). Interestingly, this phenomenon was accompanied with an increase in Td^+^ pericytes (Figure 8J), in line with elevated total Td^+^ cells. Together with the radiation data, our studies indicate a negative correlation between MALPs and vessel diameter in bone marrow.

## Discussion

**Figure 9:**
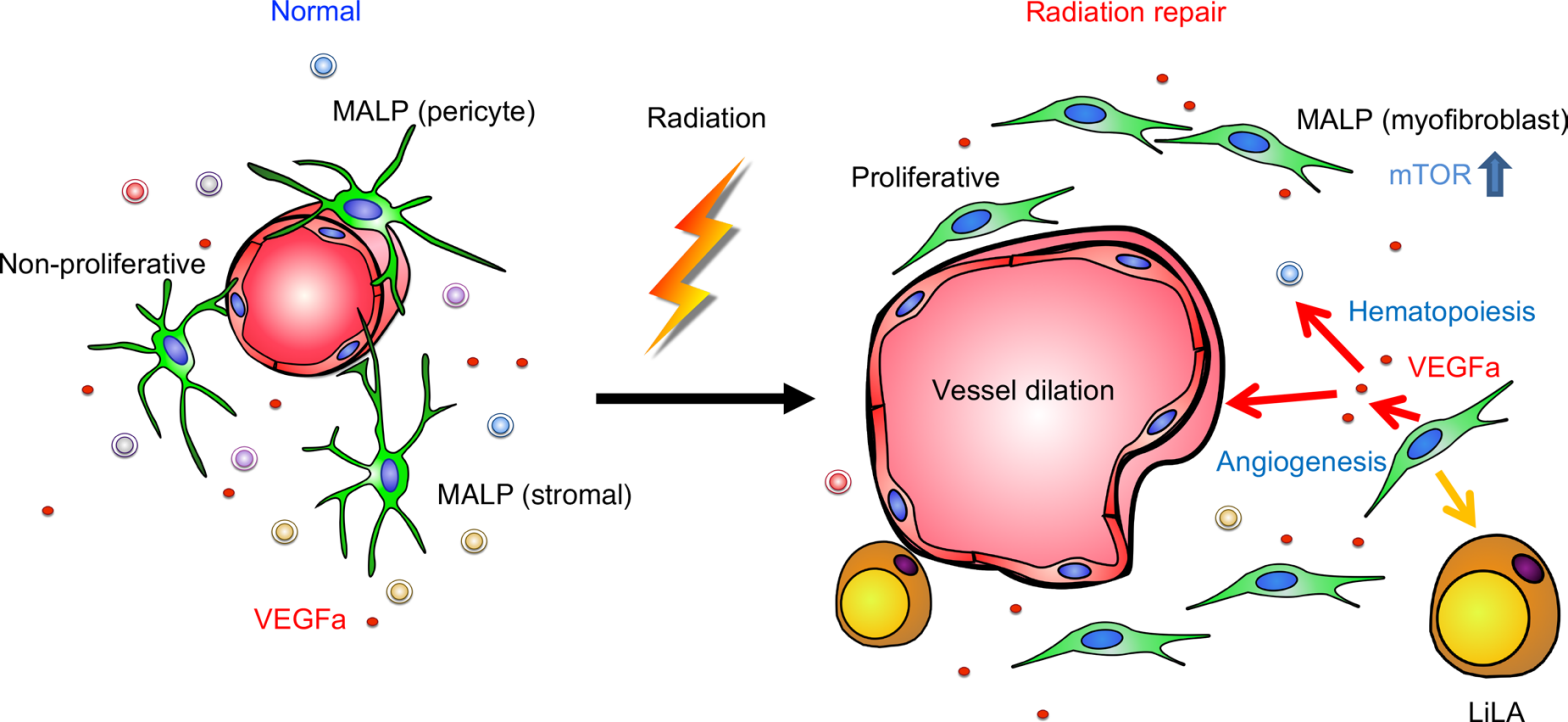
A schematic diagram depicts the role of MALPs in mediating bone marrow recovery after radiation injury.

Exposure of bone marrow to radiation can lead to local suppression of hematopoiesis at the site of irradiation at a moderate dose and bone marrow failure at a high dose due to a collapse of the hematopoietic system, including HSPCs (7, 36, 37). In the past, many studies have focused on bone marrow hematopoietic recovery after radiation, but very little attention has been paid to changes in their niche environment, i.e., mesenchymal lineage cells and vasculature in bone (38). In this study, we demonstrated that MALPs, a bone marrow adipoprecursor subpopulation we discovered previously (21), play a major role in the recovery of bone marrow hematopoietic components and vasculature. Radiation normally arrests cell cycle progression and induces cell death. To our surprise, a modest dosage of focal irradiation transformed MALPs from nonproliferative cells to proliferative cells, thus transiently increasing their number. Moreover, radiation converted MALPs into myofibroblasts, a cell type essential for wound healing and tissue regeneration, through activation of the mTOR pathway. Blocking mTOR signaling prevents the myofibroblast formation and subsequent bone marrow recovery. Our scRNA-Seq data predicted that MALPs express the highest levels of microenvironment regulatory factors such as Cxcl12, IL-7, Kitl, VEGFa, VEGFc, and Agt, which are important for hematopoiesis and angiogenesis, under normal and irradiated conditions. We subsequently validated that one of them, VEGFa, mediates the repair action of MALPs after radiation. Thus, expanding MALP population after radiation injury is an efficient method for bone marrow repair by promoting the restoration of hematopoietic components and restabilization of bone marrow capillaries (Figure 9).

The primary clinical sign of radiation damage to bone is local tissue atrophy, which means a loss of functional cells. Therefore, a rapid expansion of MALPs after radiation is a unique event. Previous studies have noticed a transient occurrence of alkaline phosphatase–positive (ALP^+^) stromal cells throughout the entire bone marrow after whole-body radiation and before marrow recovery (39, 40). Those ALP^+^ cells form a loosely meshed network interspersed among hematopoietic cells, which is very similar to the unique 3D structure formed by cell body and processes of MALPs. Interestingly, our sequencing data showed that the percentage of cells expressing *Alpl* in MALPs increased from 44.8% to 69.7% after radiation (Supplemental Figure 16), indicating that this “ALP network” is indeed made of MALPs with myofibroblast features. Another study observed an expansion of osteoblasts in mouse femurs shortly after total-body radiation (2 days) at a lethal dosage (11.25 Gy) (41). In our hands, we did not find an obvious increase in the number of osteoblasts in the radiation data set or in histology images (data not shown). Moreover, the majority of bone marrow exists in diaphyseal region with no trabecular bone. Therefore, the contribution of bone surface osteoblasts to bone marrow recovery should be minor if any in our focal radiation model. On the contrary, MALPs are ubiquitously distributed throughout the entire bone marrow. Existing abundantly as stromal cells and pericytes, they are more likely to assist the repair of hematopoietic components and capillaries.

Our scRNA-Seq analysis, EdU incorporation, and CFU-F assays all pointed out that radiation turns on the cell cycle entry of MALPs. Radiation normally damages DNA, leading to cell cycle arrest or apoptosis. Strikingly, while radiation does cause DNA DSBs in MALPs, we did not immediately observe apoptosis or cell cycle arrest. Since radiation often causes mitotic catastrophe in solid tumors (42), it is possible that MALPs undergo a few rounds of cell division before cell death. The mechanism by which radiation promotes cell proliferation in MALPs is not clear. One potential candidate is Adam17, a sheddase for growth factors such as EGF family ligands. Our single-cell sequencing data, later validated by qRT-PCR, revealed that the expression of Adam17 was highly upregulated in MALPs after radiation. This observation is consistent with previous reports that radiation increases the amount and activity of Adam17 in cancer cells, which contributes to radiation resistance of cancer (43, 44). Future experiments are required to study the role of Adam17 in MALP proliferation.

It is well known that myofibroblasts are a key player in physiological connective tissue repair after injury and in pathological fibrosis formation (45). Lineage tracing and genetic mouse models have discovered multiple precursors for myofibroblasts, including fibroblasts, pericytes, smooth muscle cells, epithelial cells, endothelial cells, and others. Myofibroblasts, identified by a set of actin filament markers, such as α-SMA, Tagln, and Vcl, and ECM components, such as type I, IV, and VIII collagen, are activated as a part of normal or dysregulated wound healing response and disappear from the normal or healed tissues. Our work here uncovers a myofibroblast conversion of MALPs after radiation. Modest radiation injury on bone marrow can be reversed without exogenous interference. However, upon severe radiation injury, we expect that newly formed myofibroblasts probably persist for a long time, leading to fibrosis. In patients and rodents, it is often observed that marrow fibrosis is accompanied by marrow ablation, suggesting a correlation between these 2 events. Interestingly, our qRT-PCR results revealed that while MALPs highly express hematopoietic regulatory factors under both normal and radiation conditions, the expression levels of these factors are actually reduced in MALPs after radiation. We hypothesize that a high dose of radiation will further diminish these factors, leading to a failure of recruiting hematopoietic cells to the bone marrow and restoring marrow vessels. Whether myofibroblast conversion is at the expense of decreased regulatory factors secreted from MALPs needs further investigation.

Our conclusion of myofibroblast conversion of MALPs is consistent with studies of primary myelofibrosis, a subtype of myoproliferative neoplasms in bone marrow. An early study reported that Lepr-expressing mesenchymal lineage cells are the source for myofibroblasts in this disease (46). A recent scRNA-Seq analysis showed that fibrosis-driving cells in ThPO-induced bone marrow fibrosis are Lepr^+^ and Adipoq^+^ mesenchymal cells (47). Single-cell transcriptomics analyses from our group and others have shown that Adipoq-expressing cells largely overlap with Lepr-expressing cells in mouse bone marrow (21, 48, 49). Thus, we believe that MALPs are the main source of bone marrow myofibroblasts and that targeting MALPs can lead to novel therapies for different types of marrow fibrosis.

MALPs are a unique type of adipocyte lineage cells because they have no counterpart in normal peripheral fat depots. In white fat tissues and brown fat tissues, *Adipoq-Cre* labels only mature, lipid-laden adipocytes (50). However, in bone marrow, it labels both lipid-free MALPs and lipid-laden LiLAs. Interestingly, a recent report studying dermal white adipose tissue found that immediately after skin injury, mature adipocytes undergo lipolysis to become proliferative, lipid-free myofibroblasts in the wound bed for subsequent repair (51). Those adipocyte-derived cells are long-lived after repair is accomplished, but they do not refill with lipids. Our studies share some similarities with this report but also have 2 important differences. First, in bone marrow, it is MALPs but not LiLAs that become proliferative myofibroblasts for tissue regeneration. Second, lipolysis is not required for the cell fate change in the bone marrow. Instead, lipid accumulation in MALPs is parallel to myofibroblast conversion.

Our scRNA-Seq analysis suggested that apart from MALPs, LCPs, the progenitor subpopulation at the lineage branch point, also exhibited increased proliferation ability and underwent myofibroblast conversion after radiation. Specifically, myofibroblast markers, such as α-SMA, Tagln, Vcl, Myl9, and so on, and biological processes, such as wound healing, stress fiber, and contractile actin filament bundle, were similarly upregulated in this cell cluster as in MALPs after radiation. Due to a lack of specific markers, we cannot further investigate their role in bone marrow repair. Nevertheless, since MALPs express hematopoietic regulatory factors and angiogenic factors at a much higher level than LCPs, we believe that MALPs are more important than LCPs in mediating marrow repair after radiation.

Altogether, our data highlight the plasticity of a type of adipogenic lineage cells in bone marrow for tissue repair. This plasticity depended on pathological conditions because aging did not convert MALPs into myofibroblasts. It would be interesting to study whether other injuries, such as chemotherapy, fracture, and marrow ablation, have similar effects on MALPs. Our further studies identify that MALP-derived VEGFa mediated marrow repair after radiation. However, blocking *Vegfa* expression in MALPs (*Vegf-CKO* mice) did not achieve the same degree of damage on bone marrow vessels and cellularity as MALP ablation (*Adipoq/DTR* mice with DT injections) after radiation, suggesting that more factors from MALPs or VEGFa from other bone marrow cells contribute to the recovery. Indeed, a recent study reported that depletion of *Vegfc*, another VEGF family member, using *Lepr-Cre* delays vascular and HSPC recovery after lethal whole-body irradiation followed by transplantation of *WT* bone marrow cells (52). Strikingly, based on our scRNA-Seq data and qRT-PCR validation, Vegfc is another angiogenic factor highly and specifically expressed in MALPs. Future lines of investigation examining additional molecular mechanisms by which myofibroblastic MALPs repair bone marrow hematopoietic components and vasculature could lead to breakthroughs in discovering treatments for untoward marrow suppression after radiotherapy or accidental exposure to high-dose radiation.

## Methods

### Animals.

*Col2a1-Cre Rosa-tdTomato* (*Col2/Td*), *Adipoq-Cre Rosa-tdTomato* (*Adipoq/Td*), and *Adipoq-CreER Rosa-tdTomato* (*AdipoqER/Td*) mice were generated by breeding *Rosa-tdTomato* mice with *Col2a1-Cre* (53), *Adipoq-Cre* (54), and *Adipoq-CreER* (55) mice, respectively. *Adipoq-Cre Rosa-tdTomato DTR* (*Adipoq/Td/DTR*) mice were generated by breeding *Adipoq/Td*, *Rosa-tdTomato*, and *Rosa-DTR* mice. *Adipoq-Cre Rosa-tdTomato Vegfa^fl/fl^* (*Vegfa-CKO*) mice were generated by breeding *Adipoq/Td*, *Rosa-tdTomato*, and *Vegfa^fl/fl^* mice. All mouse lines have a C57BL/6 background and were purchased from The Jackson Laboratory, except that *Vegfa^fl/fl^* mice were obtained from Genentech. For most radiation experiments, the right femurs of 1- to 2-month-old male mice received 5 Gy radiation at a rate of 1.65 Gy/min from a focal irradiator (SARRP, Xstrahl), using an adjusted collimator with the aid of onboard cone beam computed tomography and x-ray as described previously (56). The final size of the collimator was 5 mm in width and 15 mm in length to cover the entire femur but no other body parts. For lineage tracing experiments, mice received tamoxifen injections (MilliporeSigma, 75 mg/kg/d) at indicated ages and their bones were harvested later. For rapamycin treatment, *Adipoq/Td* mice received intraperitoneal vehicle (1× PBS) or rapamycin (MilliporeSigma, 4 mg/kg) injections 1 day before radiation and injections every other day after radiation. Bones were harvested at days 3 and 14 for histology analysis. For cell ablation experiments, *Adipoq/Td/DTR* mice received 5 Gy SARRP radiation to both femurs followed by vehicle (1× PBS) or DT injections (50 μg/kg) every other day for 2 weeks.

### Endosteal bone marrow Td^+^ cell isolation and cell sorting.

Endosteal bone marrow cells were harvested as described previously (57). Briefly, the outer surfaces of long bones were scraped and digested to remove the periosteum. After cutting off the epiphyses and flushing out the central bone marrow, metaphyseal bone fragments were longitudinally cut into 2 halves and digested by proteases to collect endosteal bone marrow cells. Freshly isolated endosteal bone marrow cells were resuspended in FACS buffer containing 25 mM HEPES (Thermo Fisher Scientific) and 2% FBS in PBS and sorted for top 1% Td^+^ cells if a Td peak was not obvious or Td^+^ cells if a Td peak was obvious using Influx B (BD Biosciences) or Aria B (BD Biosciences).

### ScRNA-Seq of endosteal bone marrow cells.

We constructed 3 batches of single-cell libraries for sequencing: endosteal Td^+^ bone marrow cells from 1-month-old mice (*n* = 2), 1.5-month-old mice (*n* = 3), and 1-month-old mice with radiation (*n* = 3). A total of 20,000 cells were loaded with the aim of acquiring 1 single library of 10,000 cells for each age group by Chromium controller (V2 chemistry version, 10x Genomics), barcoded and purified as described by the manufacturer, and sequenced using a 2 × 150 paired-end configuration on an Illumina HiSeq platform at a sequencing depth of about 400 million reads. Cell Ranger (Version 3.0.2, https://support.10xgenomics.com/single-cell-gene-expression/software/pipelines/latest/what-is-cell-ranger) was used to demultiplex reads, followed by extraction of cell barcode and UMIs. The cDNA insert was aligned to a modified reference mouse genome (mm10). Detailed computational analyses of data sets are described in Supplemental Methods.

### Histology.

To obtain whole-mount sections for immunofluorescence imaging, freshly dissected bones were fixed in 4% paraformaldehyde for 1 day, decalcified in 10% EDTA for 4–5 days, and then immersed into 20% sucrose and 2% polyvinylpyrrolidone (PVP) at 4°C overnight. Samples were embedded into 8% gelatin in 20% sucrose and 2% PVP embedding medium and sectioned at 50 μm in thickness. Sections were incubated with rat anti-CD45 (BioLegend, 103101), rat anti-Endomucin (Santa Cruz Biotechnology, sc-65495), rabbit anti-Perilipin (Cell Signaling Technology, 9349), rabbit anti-mouse Collagen IV (Abcam, ab6586), mouse anti-αSMA (MilliporeSigma, A2547), rabbit anti-Tagln (Proteintech, 10493-1-AP), rabbit anti-Myl9 (Proteintech, 15254-1-AP), rabbit anti-γH2AX (Cell Signaling Technology, 9718s), rabbit anti–phospho–4E-BP1 (Cell Signaling Technology, 2855S), and rat anti-CD150 (BioLegend, 115901) antibodies at 4°C overnight, followed by Alexa Fluor 488 donkey anti-rat (Life Technologies, A-21208), Alexa Fluor 488 goat anti-mouse (Life Technologies, A-11029), Alexa Fluor 488 donkey anti-rabbit (Life Technologies, A-21206), or Alexa Fluor 647 donkey anti-rabbit (Life Technologies, A-21246) secondary antibody incubation 1 hour at room temperature. For EdU staining, mice received 1.6 mg/kg EdU 1 day and 3 hours before sacrifice, and the staining was carried out according to the manufacturer’s instructions (Thermo Fisher Scientific, Click-iT EdU Alexa Fluor 647 Imaging Kit, D3822). The TUNEL assay was carried out according to the manufacturer’s instructions (MilliporeSigma, s7101). Lipid was stained by BODIPY FL C12 according to the manufacturer’s instructions (Thermo Fisher Scientific, D-3822).

To reconstruct 3D structure of bone marrow, fluorescence images were captured by a Zeiss LSM 710 scanning confocal microscope interfaced with the Zen 2012 software (Carl Zeiss). Confocal image stacks were collected to a depth of approximately 50 μm and a step size of 1 μm at 63× original magnification. Laser power and detector sensitivity were adjusted for *Z*-correction to compensate for signal dissipation at greater imaging depths. Detector gain and offset were adjusted according to the most intense regions to ensure minimal saturation of the signal over the entire imaging area. Imaris 9.2 software (Oxford Instruments) was used for processing *Z*-stacks. To characterize bone marrow vessels, all vessels in a 0.3 mm^2^ area in the diaphyseal bone marrow were selected to measure their diameter of axis (vessel diameter), area (vessel area), and number (vessel density). The number of cell processes per cell was manually quantified. Cell circularity was quantified by ImageJ (NIH; https://imagej.nih.gov/ij/). A circularity value of 1.0 indicates a perfect circle. As the value approaches 0.0, it indicates an increasingly elongated polygon.

### Cytokine assay.

Bone marrow was pelleted via centrifugation and lysed in RIPA buffer with proteinase inhibitor (MilliporeSigma). The supernatant of lysed samples was used for measuring cytokine amounts, including VEGFa, using Proteome Profiler Mouse XL Cytokine Array (R&D Systems, ARY028), normalized by total protein amount according to manufacturer’s instructions.

### Cell culture.

To count CFU-F number, flushed bone marrow cells were plated at 3 × 10^6^ cells/T25 flask in growth medium (α-MEM supplemented with 15% FBS, 0.1% β-mercaptoethanol, 20 mM glutamine, 100 IU/mL penicillin, and 100 μg/mL streptomycin) for 7 days. Confluent mesenchymal progenitors were switched to adipogenic medium (DMEM with 10% FBS, 10 ng/mL triiodothyronine, 1 μM rosiglitazone, 1 μM dexamethasone, 10 μg/mL insulin, 100 IU/mL penicillin, and 100 μg/mL streptomycin) for 7 days. Bright-field and fluorescence images of cell culture were captured by fluorescence inverted microscopy (Nikon Eclipse, TE2000-U).

### qRT-PCR analysis.

Sorted or cultured cells were collected in TRIzol Reagent (MilliporeSigma). A Taqman Reverse Transcription Kit (Applied Biosystems) was used to reverse-transcribe mRNA into cDNA. Following this, qRT-PCR was performed using a Power SYBR Green PCR Master Mix Kit (Applied Biosystems). The primer sequences for the genes used in this study are listed in Supplemental Table 1.

### Hematopoietic phenotyping of bone marrow cells.

PB of mice was collected retro-orbitally, and hematopoietic parameters were measured by complete blood counts. Central bone marrow was flushed from femurs, and cellularity was quantified with 3% acetic acid in methylene blue (STEMCELL Technologies, 07060). For flow cytometric analysis of the lineage cell compartment of mice, flushed bone marrow cells were stained for myeloid (rat anti–Gr-1 APC-Cy7, BD Biosciences, 557661; rat anti–Mac-1 APC, eBioscience, 17-0112-83) or lymphoid lineages (rat anti-B220 FITC, eBioscience, 11-0452-82; hamster anti-CD3 PE-Cy7, eBioscience, 25-0031-82). The HSPC compartment was analyzed by staining for Lineage (biotin–Ter-119, –Mac-1, –Gr-1, -CD4, -CD8α, -CD5, -CD19, and -B220, eBioscience, 13-5921-85, 13-0051-85, 13-5931-86, 13-0112-86, 13-0452-86, 13-0041-86, 13-0081-86, 13-0193-86) followed by staining with streptavidin–PE-Texas Red (Invitrogen, SA1017), rat anti-cKit APC-Cy7 (eBioscience, 47-1171-82), rat anti-Sca1 PerCP-Cy5.5 (eBioscience, 45-5981-82), hamster anti-CD48 APC (eBioscience, 17-0481-82), and rat anti-CD150 PE-Cy7 (BioLegend, 115914). All flow cytometry analysis was performed on a BD LSR Fortessa flow cytometer with FlowJo v10.7.1 for Mac.

### Availability of data.

Sequencing data have been deposited in National Center for Biotechnology Information’s Gene Expression Omnibus under accession codes GSE145477 and GSE166129.

### Statistics.

Data are represented as mean ± SD and analyzed by 2-tailed Student’s *t* tests or 1-way or 2-way ANOVA with Tukey’s posttest for multiple comparisons using Prism software (GraphPad Software). For cell culture experiments, observations were repeated independently at least 3 times with a similar conclusion, and only data from a representative experiment are presented. Values of *P* < 0.05 were considered significant.

### Study approval.

All animal work performed in this report was approved by the Institutional Animal Care and Use Committee at the University of Pennsylvania.

## Author contributions

LZ, LY, and LQ designed the study. LZ, LY, and YE performed animal experiments. LZ performed histology and imaging analysis. NH performed flow cytometry analysis of hematopoietic cells. LZ, LY, WY, and TG performed cell culture and qRT-PCR experiments. LY and ZM performed computational analyses. ML, KSJ, YG, AM, TMB, MP, KC, PS, and WT provided administrative, technical, or material support and consultation. LQ wrote the manuscript. LZ, LY, KJ, WT, MP, and PS reviewed and revised the manuscript. LQ approved the final version. LZ and LY contributed equally to this project.

## Supplementary Material

Supplemental data

## Version 1 04/08/2022

Electronic publication
