# Supplementary material for: Transient expansion and myofibroblast conversion of adipogenic lineage precursors mediate bone marrow repair after radiation
Source: JCI Insight. 2022 Apr 8;7(7):e150323. doi: 10.1172/jci.insight.150323 (PMC9057603; doi:10.1172/jci.insight.150323)
Supplement: Supplemental data [file jciinsight-7-150323-s051.pdf]

Supplementary Methods

Computational analysis of scRNA-seq data

Seurat package V3 (60) was used for individual or integrated analysis of datasets. Standard Seurat pipeline was used for filtering, variable gene selection, dimensionality reduction analysis and clustering. Doublets or cells with poor quality (genes>6000, genes<200, or >5% genes mapping to mitochondrial genome) were excluded. Expression was natural log transformed and normalized for scaling the sequencing depth to a total of $1x10^4$ molecules per cell. For the integrated dataset, anchors from different dataset were defined using the FindIntegrationAnchors function, and these anchors were then used to integrate datasets together with IntegrateData. For individual dataset analysis, we first identify variable genes by controlling for the relationship between average expression and dispersion and then scale integrated or individual datasets by regressing out the number of UMIs and percent mitochondrial genes. Statistically significant principal components (PC) were selected as input for uniform manifold approximation and projection (UMAP) plots. Different resolutions for clustering were used to demonstrate the robustness of clusters. Sub-clustering was performed by isolating the mesenchymal lineage clusters using known marker genes, followed by reanalysis as described above. Seurat Cell-cycle scoring function were used to analyze cell proliferation. Proliferative cells were defined as cells in G2M or S Phase, following by regressing out the Cell-cycle heterogeneity effect and restart from finding significant PCs and dimensional reduction. Differentially expressed genes between normal and radiated groups were identified using FindMarkers within Seurat and p values between them were calculated by "test.use=bimod". Hierarchy analysis was performed using BuildClusterTree function. Gene ontology analysis and GSEA was performed using the ClusterProfiler package (61).

Computational cell cycle analysis was conducted as described previously (21), we used a core set of 43 S and 54 G2/M genes defined previously (62). First, the genes that are expressed in less than 5% of total cells were removed, resulting in 34 S and 45 G2/M genes for the following cell cycle analysis. Second, we define proliferative cells if the cell express S gene set or G2/M gene set, that is, there is a significant difference between the expression value of S genes and G2/M genes. Since the null distribution for the S gene set and G2/M gene set is unknown, we designed a permutation test which does not assume a null-distribution. To be more specific, for each cell, we resampled 34 genes from the 79 genes (34+45) as "S genes" and the rest 45 genes as "G2/M genes". This resampling was repeated 5000 times and each time, we calculate the difference between the mean expression value of "S genes" and the mean expression values of "G2/M genes". The original difference between the mean expression value of S genes and the mean expression value of G2M was compared with the difference between resampling results to get a significant score (p-values). To correct for multi-test, FDR corrections were applied to each cluster. At last, FDR p-value < 0.05 was used as a cutoff for distinguishing proliferative or non-proliferative cells.

To computationally delineate the developmental progression of bone marrow mesenchymal cells and order them in pseudotime, we used the algorithms implemented in the Monocle 2 package (63). We include mesenchymal lineage cells with no chondrocytes from separated or integrated dataset of different group (NR or R) mice for the analysis. We decided the genes that define the cell differentiation trajectory by selecting genes with high dispersion across cells, using a parameter of “mean_expression >= 0.05 & dispersion_empirical >= 2 * dispersion_fit”. The gene list was further used for dimensional reduction to generate the trajectory reconstruction using the nonlinear reconstruction algorithm DDRTree.

We also performed the trajectory analysis using Slingshot (64). Briefly, UMAP was used as dimensional reduction after the PCA were calculated for individual or integrated datasets. Then Seurat objects were transformed into SingleCellExperiment objects. Slingshot trajectory analysis were conducted using the Seurat clustering information and with dimensionality reduction produced by UMAP.

The SCENIC algorithm was used to assess the regulatory network analysis regard to transcription factors (TFs) and discover regulons (TFs and their target genes) in individual cells. Following the standard pipeline, the gene expression matrix with gene names in rows and cells in columns was input to SCENIC (version 0.9.1) (65). The genes were filtered with default parameter, and co-expressed genes for each TF were constructed with GENIE3 software, followed by calculating of Spearman's correlation between TFs and their potential targets, and then the "runSCENIC" procedure assisted to generate the GRNs (also termed regulons). Finally, regulon activity was analyzed by AUCell (Area Under the Curve) software. Regulon activity of each cell was input back to Seurat object and increased or decreased regulons in MALPs were visualized by heatmap.

Supplementary Figures

Figure S1

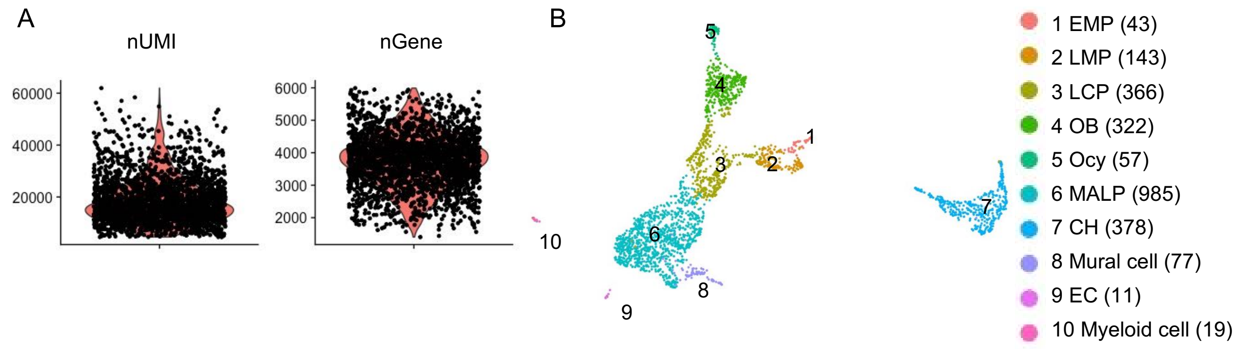

Figure S1. Large scale scRNA-seq analysis of bone marrow Td^+ cells from 1-month-old *Col2/Td* mouse femurs at day 3 post focal radiation.

(A) Violin plots show numbers of UMIs and genes per cell in the irradiated dataset.

(B) The UMAP plot of 2401 sequenced endosteal bone marrow cells (n = 5 mice). Cell numbers are listed in parenthesis next to cluster names. EMP: early mesenchymal progenitor; LMP: late mesenchymal progenitor; LCP: lineage committed progenitor; OB: osteoblast; Ocy: osteocyte; MALP: marrow adipogenic lineage precursor; CH: chondrocyte; EC: endothelial cells.

Figure S2

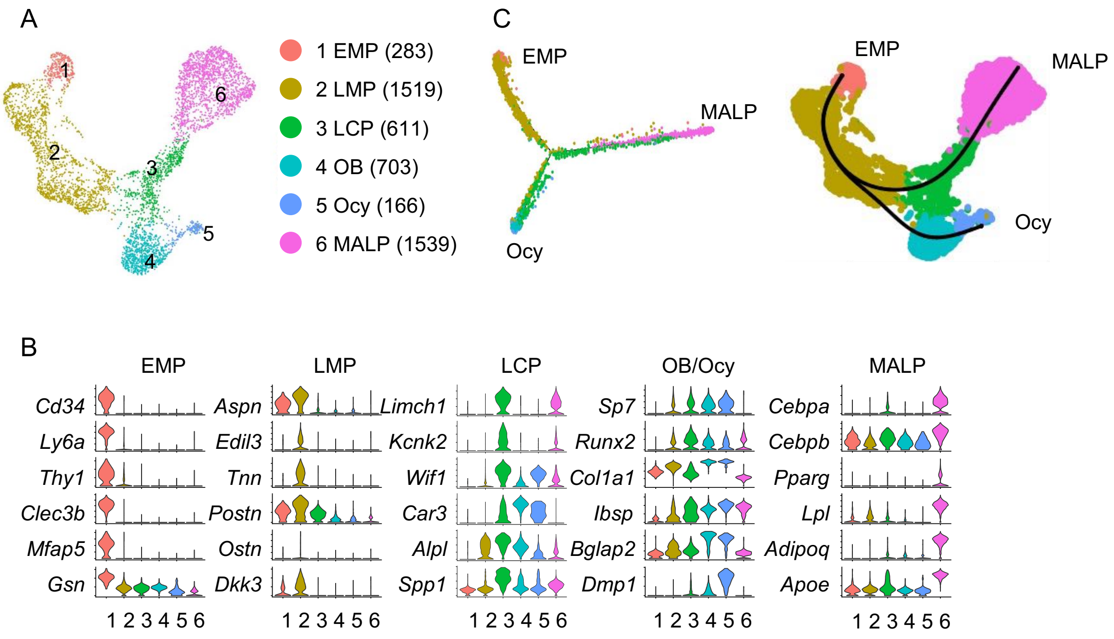

Figure S2. Large scale scRNA-seq analysis of bone marrow Td^{+} cells from 1-1.5-month-old normal *Col2/Td* mouse femurs.

(A) The UMAP plot of 4821 Td^{+} mesenchymal lineage cells (n = 5 mice). Cell numbers are listed in parenthesis next to cluster names. EMP: early mesenchymal progenitor; LMP: late mesenchymal progenitor; LCP: lineage committed progenitor; OB: osteoblast; Ocy: osteocyte; MALP: marrow adipogenic lineage precursor.

(B) Violin plots of marker gene expression for indicated cell clusters.

(C) Monocle (left) and slingshot (right) trajectory plots of sequenced cells.

Figure S3

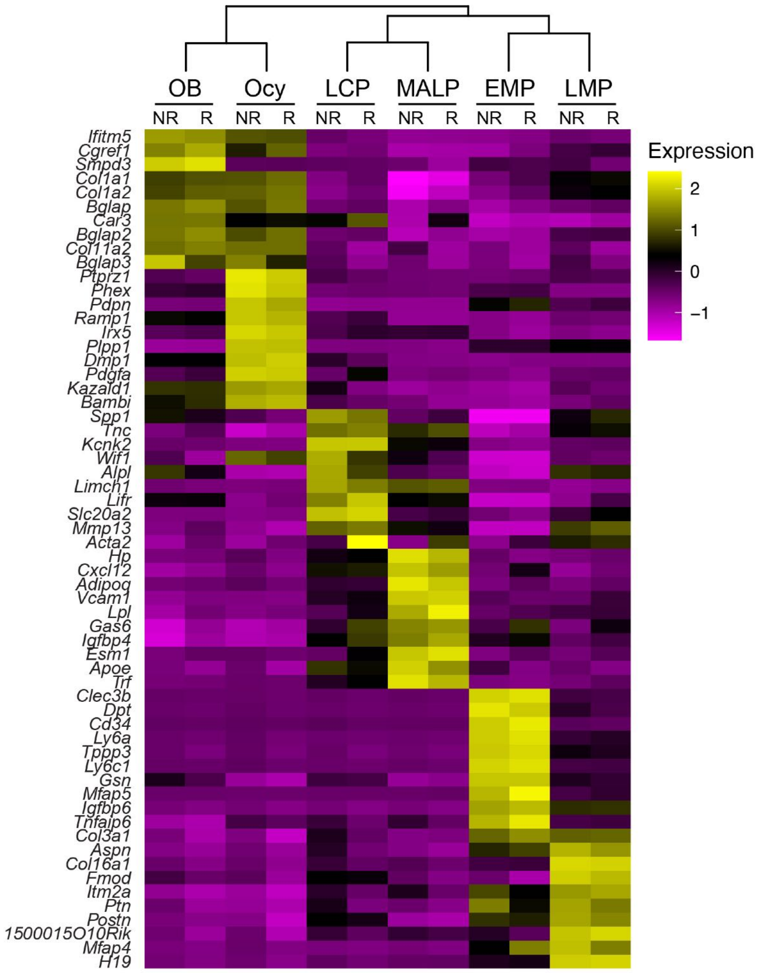

Figure S3. Hierarchy analysis of mesenchymal lineage cell clusters in the integrated scRNA-seq dataset containing normal (NR) and irradiated (R) cells. Top 10 genes ranked by avg_log2FC of each cluster are listed.

Figure S4

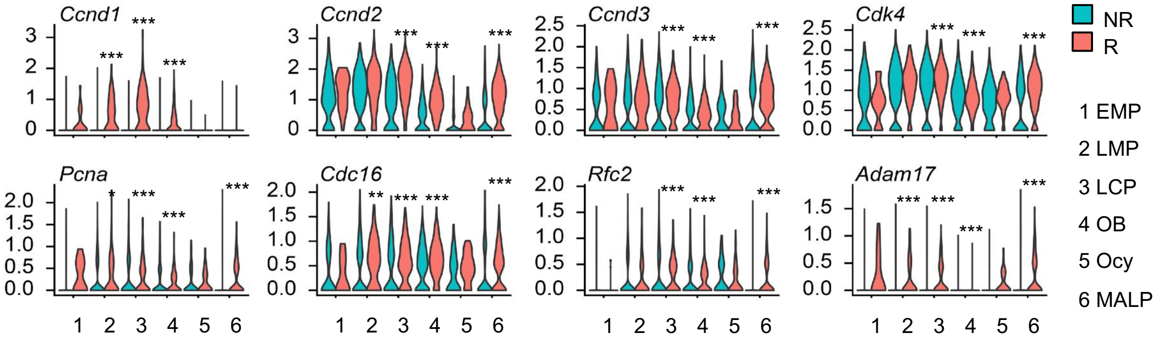

Figure S4. Violin plots of genes promoting cell proliferation.

Statistical analysis was performed using “bimod” test.use in Findmarkers function. *: p<0.05; **: p<0.01; ***: p<0.001.

Figure S5

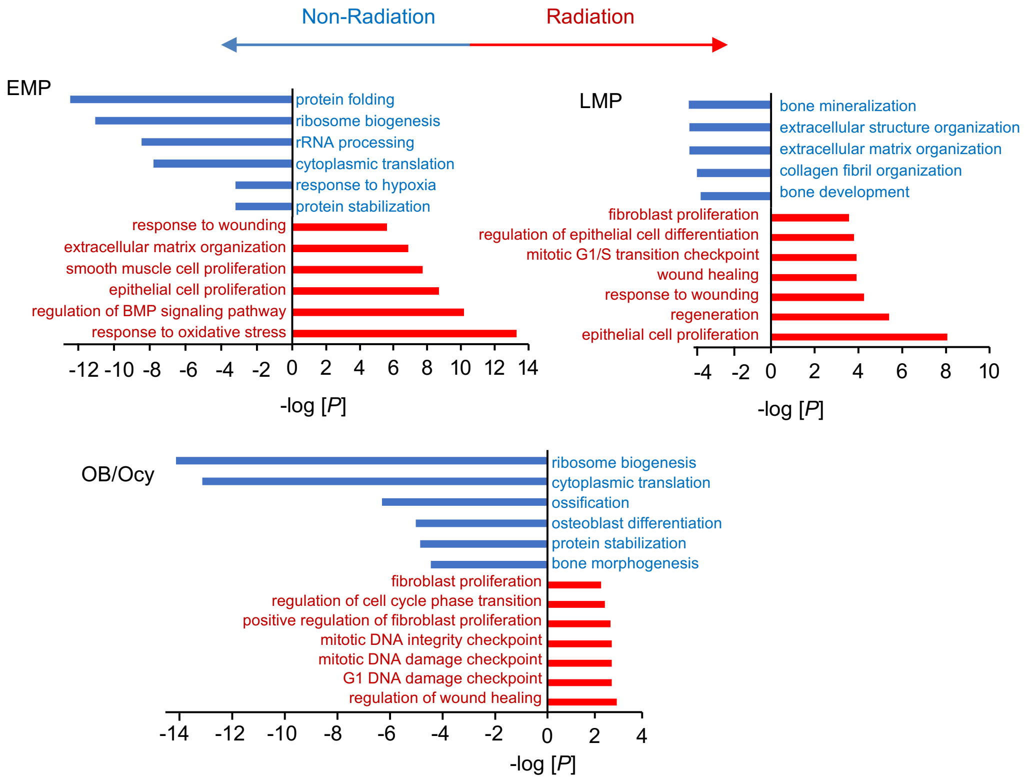

Figure S5. GO term and KEGG pathway analyses of genes differentially regulated in EMPs, LMPs, and osteoblasts/osteocytes (OB/Ocy) after radiation.

Figure S6

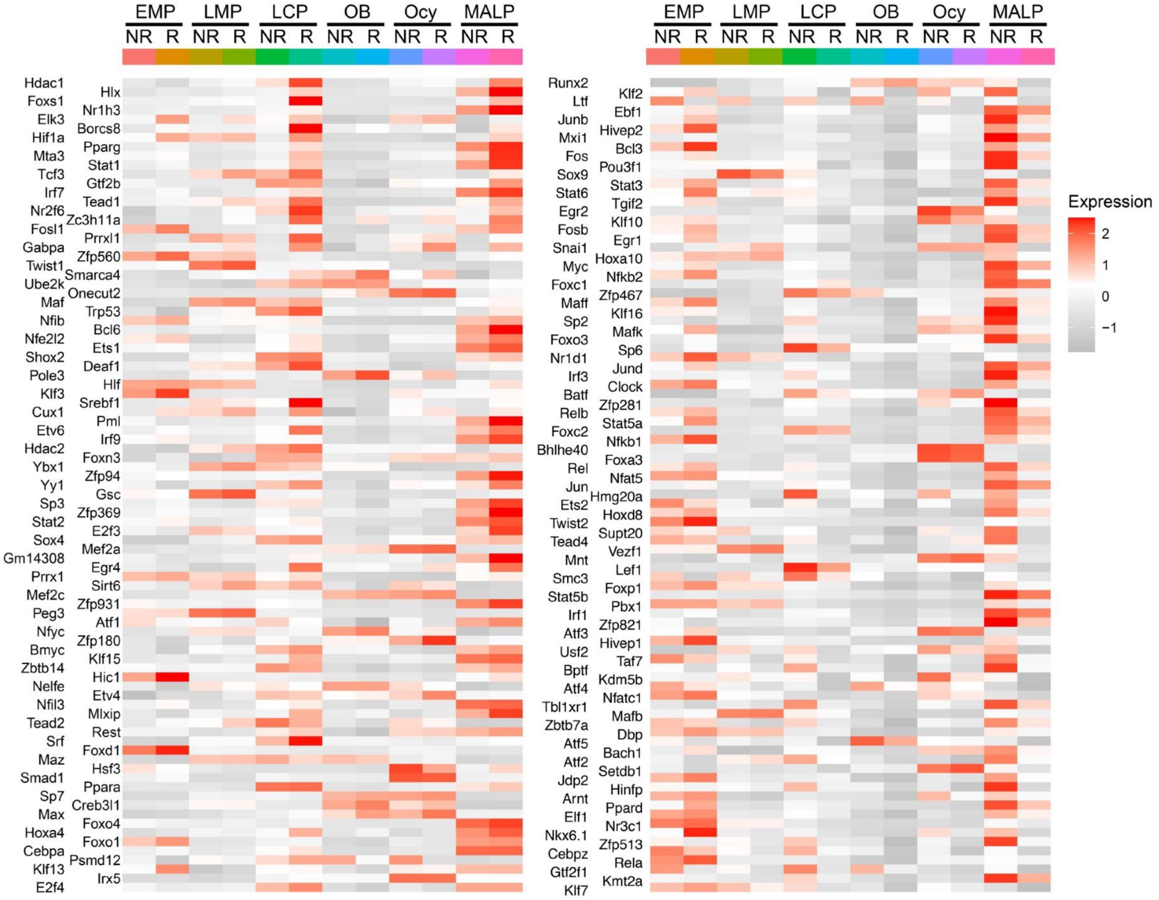

Figure S6. Regulon analysis of integrated datasets containing normal (NR) and irradiated (R) cells reveals the transcription factors whose activities are either up-regulated (left) or down-regulated (right) in MALPs after radiation.

Figure S7

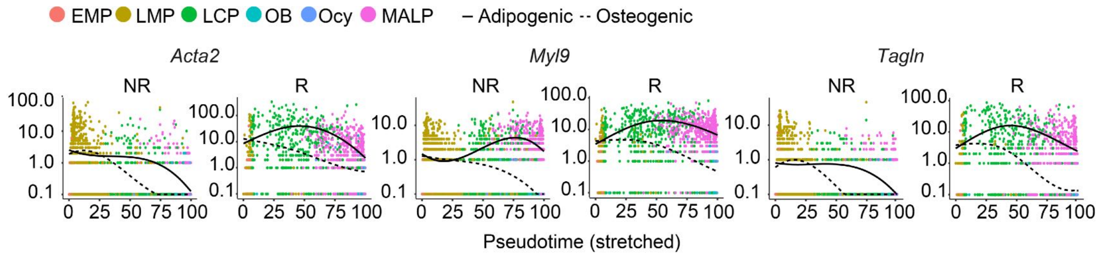

Figure S7. Expression of myofibroblast marker genes goes from the progenitor state and bifurcates into osteogenic or adipogenic branches with respect to pseudotime coordinates in normal (NR) and irradiated (R) datasets.

Figure S8

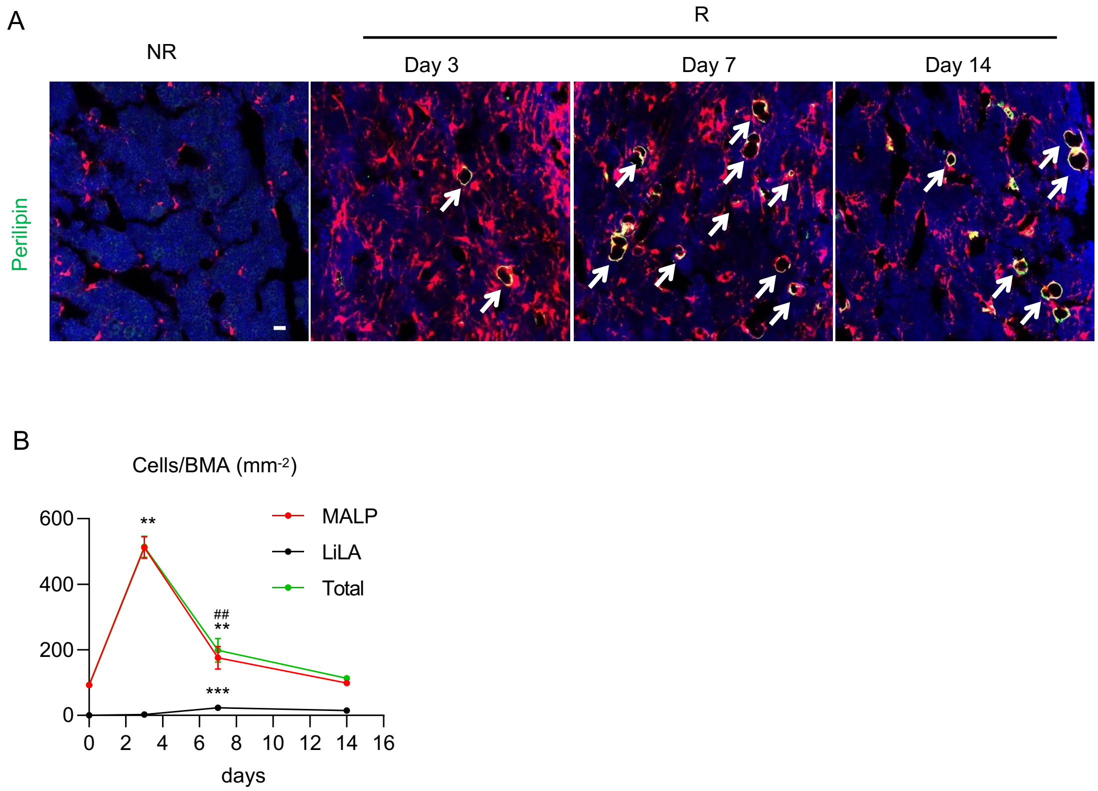

Figure S8. MALPs are precursors for radiation-induced LiLAs.

(A) Representative fluorescent images of *AdipoqER/Td* femoral bone marrow with Perilipin staining before (NR) and after (R) focal radiation. Scale bar: 20 µm.

(B) Quantification of MALPs, LiLAs, and total adipogenic lineage cells (MALPs plus LiLAs) in *AdipoqER/Td* femora bone marrow after radiation. n = 3 mice/group.

One-Way ANOVA with Tukey's multiple-comparison test. *: p<0.05; **: p<0.01; ***: p<0.001.

Figure S9

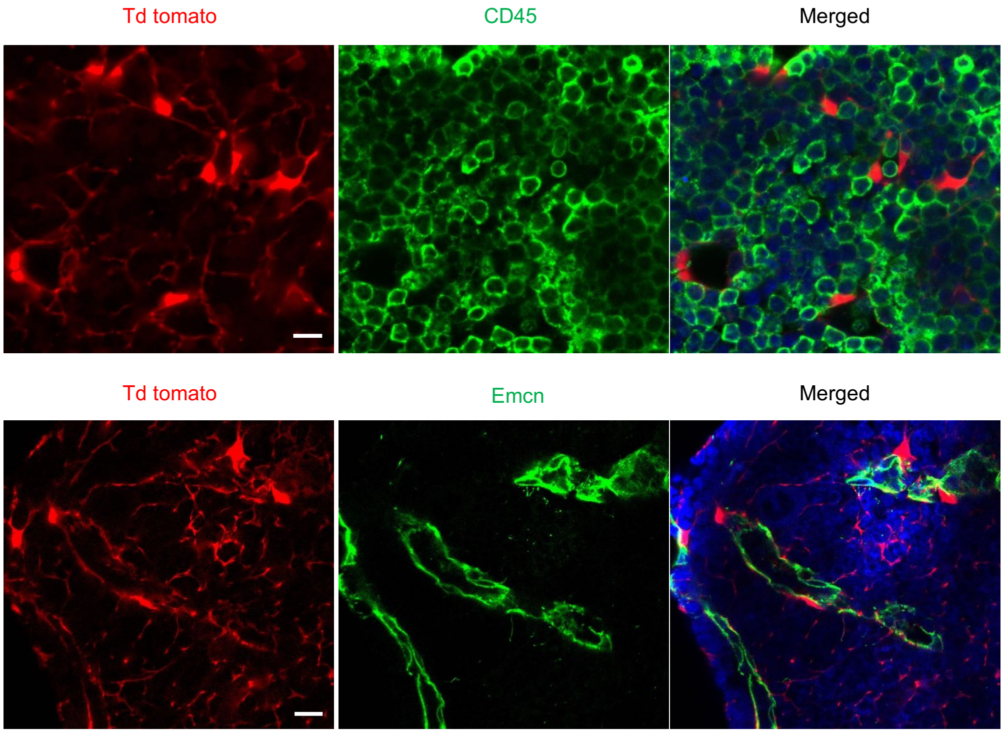

Figure S9. Immunofluorescent staining shows that Td signal in the bone marrow of *Adipoq/Td* mice do not overlap with $Cd45^+$ hematopoietic cells (top panel) or $Emcn^+$ endothelial cells (bottom panel). Scale bar: 10 μm (top) and 20 μm (bottom).

Figure S10

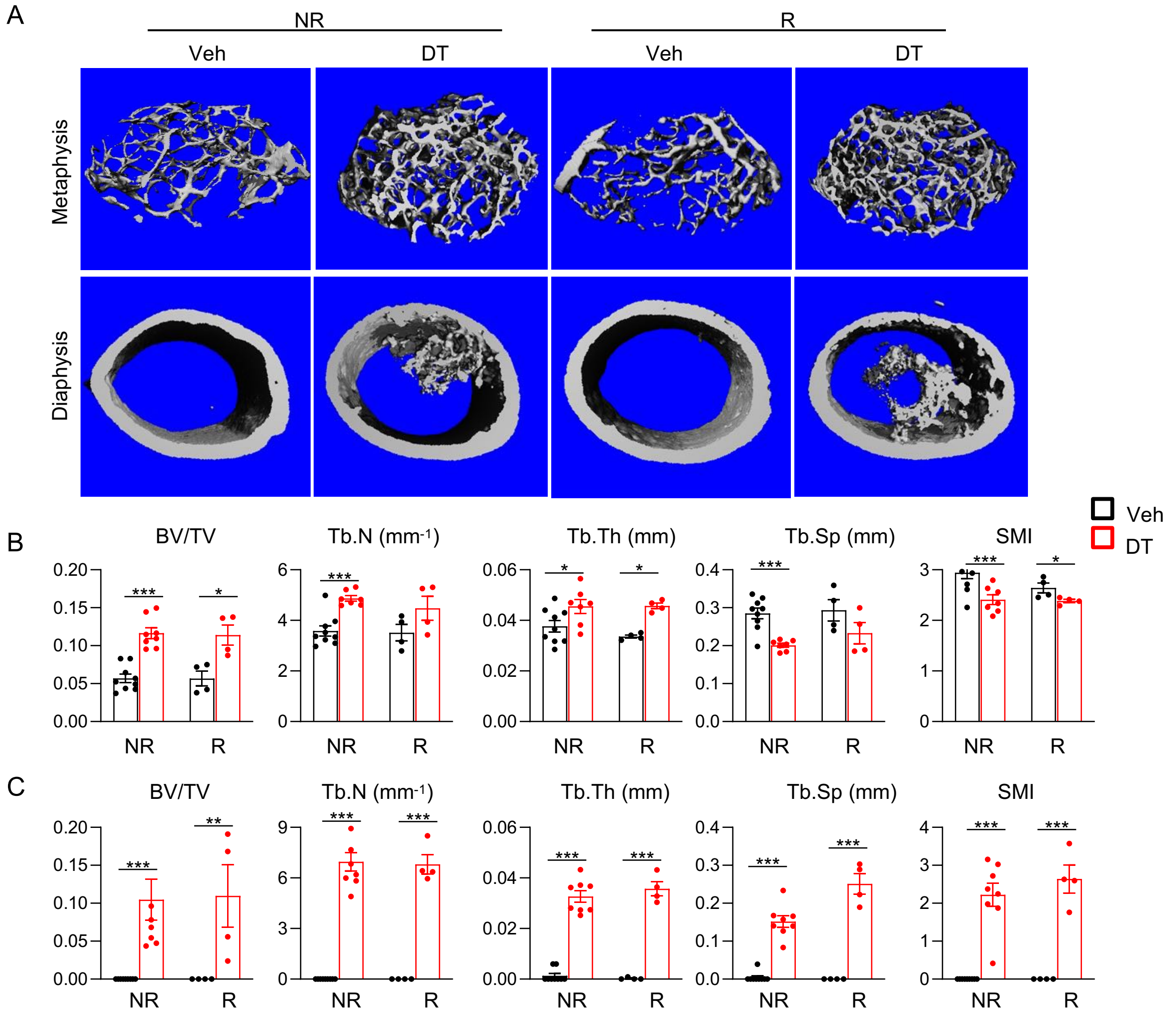

Figure S10. MALP ablation increases bone mass in both non-irradiated and irradiated mice. *Adipoq/Td/DTR* mice at 1 month of age received 5Gy focal radiation to their right femurs, followed by vehicle or DT injections every other day. Bones were harvested at day 14 for microCT analysis.

(A) Representative 3D microCT images of metaphyseal trabecular bone (top) and diaphyseal cortical and trabecular bone (bottom).

(B) Quantification of metaphyseal trabecular bone structural parameters.

(C) Quantification of diaphyseal trabecular bone structural parameters.

BV/TV: bone volume fraction; Tb.N: trabecular number; Tb.Th: trabecular thickness; Tb.Sp: trabecular separation; SMI: structural model index. n=4-9 mice/group.

Statistical analysis was performed using one-way ANOVA with Tukey's multiple-comparison analysis. *: $p<0.05$; **: $p<0.01$; ***: $p<0.001$.

Figure S11

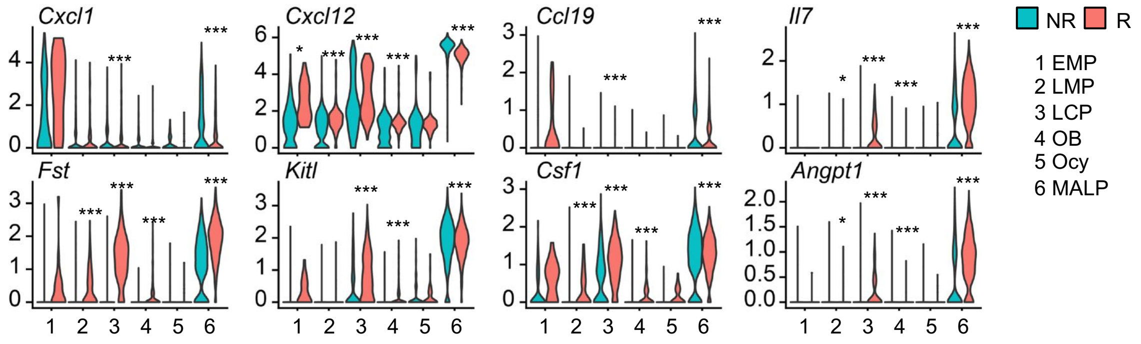

Figure S11. MALPs express hematopoietic regulatory factors at the highest level among bone marrow mesenchymal lineage cells.

Violin plots show the expression patterns of those factors in normal (NR) and irradiated (R) scRNA-seq datasets.

Statistical analysis was performed using “bimod” test.use in Findmarkers function. *: $p<0.05$; **: $p<0.01$; ***: $p<0.001$.

Figure S12

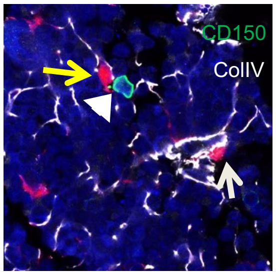

Figure S12. MALPs provide niches for HSPCs.

Immunofluorescent staining of bone marrow from *Adipoq/Td* mice shows a $CD150^{+}$ HSCP (pointed by a white arrowhead) resides next to a Td^{+} cell (pointed by a yellow arrow). ColIV is a marker for both endothelial cells and MALPs. A white arrow points to a pericytic Td^{+} cell.

Figure S13

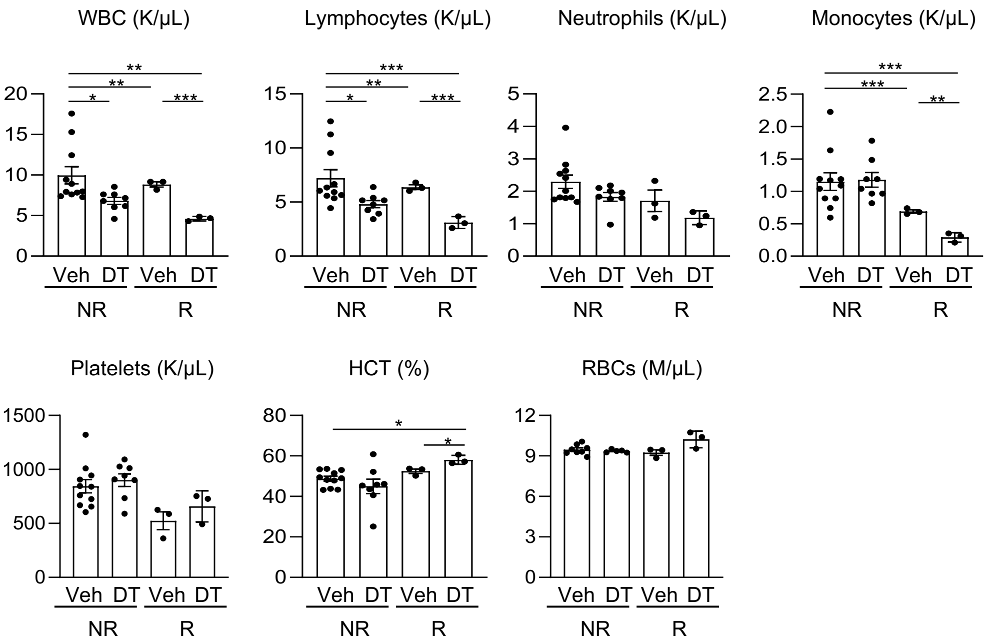

Figure S13. MALP ablation mitigates the recovery of peripheral blood after radiation.

Flow analysis of peripheral blood components, such as white blood cells (WBCs), neutrophils, lymphocytes, monocytes, platelets, hematocrit (HCT), and red blood cells (RBCs) in *Adipoq/Td/DTR* mice receiving 2 weeks of vehicle (Veh) or DT injections with or without prior radiation. n = 3-11 mice/group.

One-Way ANOVA with Tukey's multiple-comparison test. NR: non-irradiated; R: irradiated. *: $p<0.05$; **: $p<0.01$; ***: $p<0.001$.

Figure S14

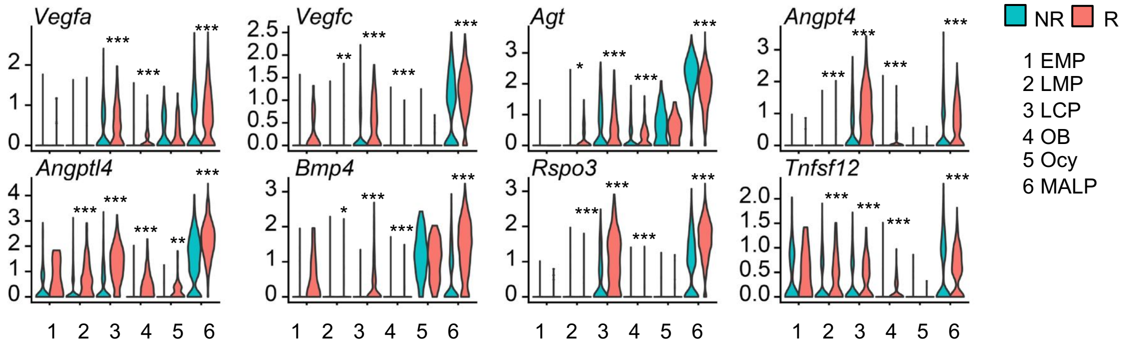

Figure S14. MALPs express angiogenic factors at the highest level among bone marrow mesenchymal lineage cells.

Violin plots show the expression patterns of those factors in normal (NR) and irradiated (R) scRNA-seq datasets.

Statistical analysis was performed using "bimod" test.use in Findmarkers function. *: $p<0.05$; **: $p<0.01$; ***: $p<0.001$.

Figure S15

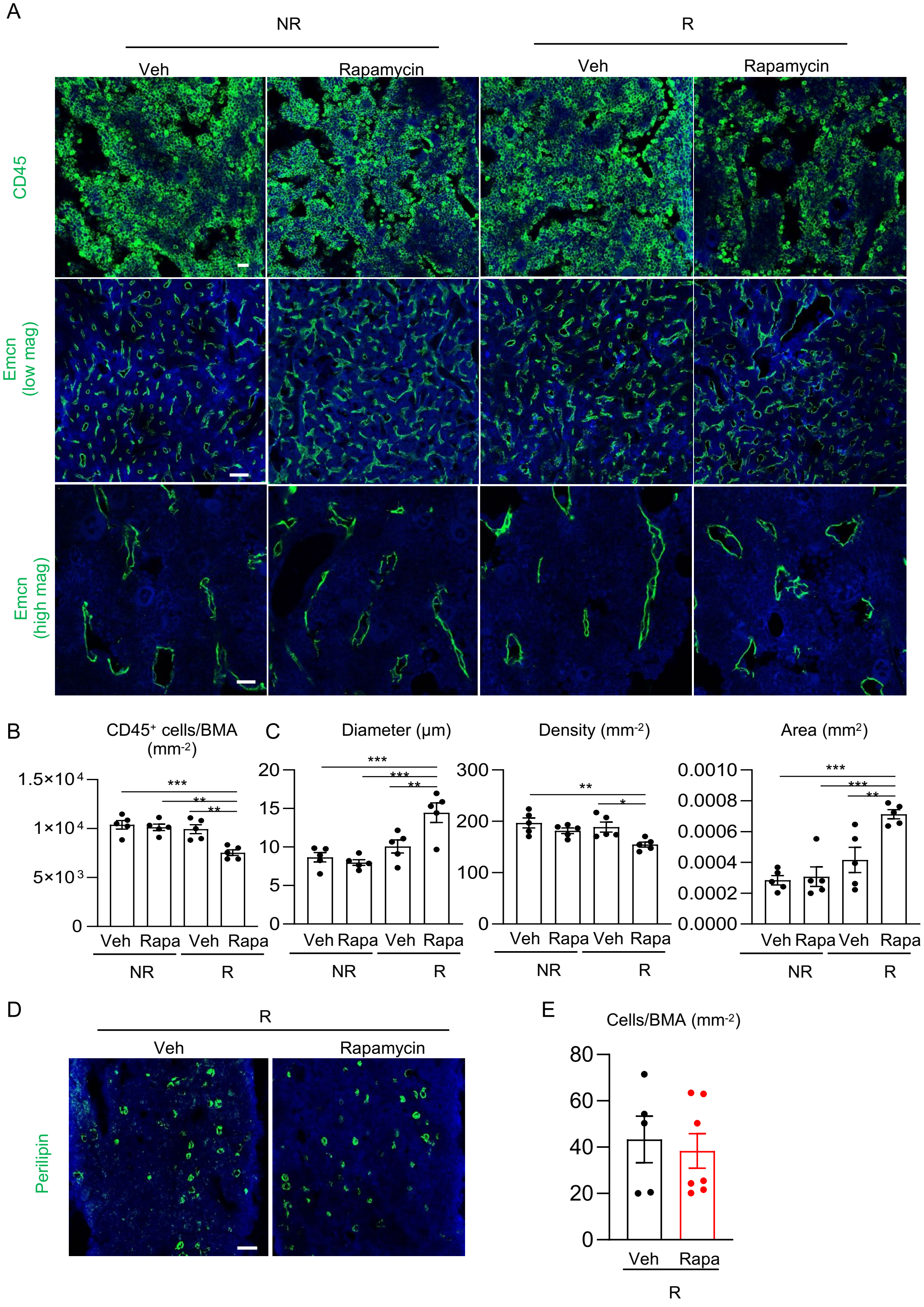

Figure S15. Rapamycin treatment after radiation prevents bone marrow recovery but not marrow

adiposity.

(A) Representative fluorescent images of *Adipoq/Td* femoral bone marrow with CD45 and Emcn staining. Mice received intraperitoneal vehicle (1xPBS) or rapamycin (4 mg/kg) injections one day before radiation and every other day injection after radiation. Bones were harvested at day 14 for analysis. Scale bar: 20 µm (top), 100 µm (middle) and 20 µm (bottom).

(B) $CD45^+$ hematopoietic cells were quantified in bone marrow. BMA: bone marrow area. n = 5 mice/group.

(C) Vessel diameter, density, and area were quantified in bone marrow (n = 5). n = 5 mice/group.

(D) Representative fluorescent images of Perilipin staining. Scale bar: 100 µm.

(E) $Perilipin^+$ LiLAs were quantified. BMA: bone marrow area. n = 5 mice/group.

Statistical analysis was performed using one-way ANOVA with Tukey's multiple-comparison analysis for panel B and C; Nonparametric Student's t test was used for panel E. *: $p<0.05$; **: $p<0.01$; ***: $p<0.001$.

Figure S16

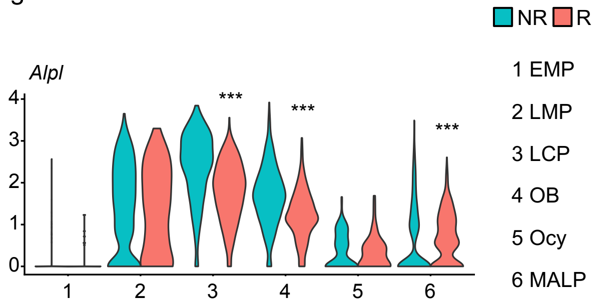

Figure S16. Radiation rapidly increases *Alpl* expression in MALPs.

Violin plots show the expression patterns of *Alpl* in normal (NR) and irradiated (R) scRNA-seq datasets.

Statistical analysis was performed using "bimod" test.use in Findmarkers function. *: p<0.05; **: p<0.01; ***: p<0.001.

Supplementary Table 1

Mouse real time RT-PCR primer sequences used in this study.

Gene	Forward primer	Reverse primer
Ccnd1	5'-GCGTACCCTGACACCAATCTC-3'	5'-CTCCTCTTCGCACTTCTGCTC-3'
Ccnd2	5'-GAGTGGGAACTGGTAGTGTTG-3'	5'-CGCACAGAGCGATGAAGGT-3'
Ccnd3	5'-CGAGCCTCCTACTTCCAGTG-3'	5'-GGACAGGTAGCGATCCAGGT-3'
Cdk4	5'-CTGAACCGCTTTGGCAAGAC-3'	5'-GCCCTCTCTTATCGCCAGAT-3'
Adam17	5'-AGGACGTAATTGAGCGATTTTGG-3'	5'-TGTTATCTGCCAGAAACTTCCC-3'
Pparg	5'-CCAGCGTGAAGCCAGAGTAG-3'	5'-ACCGTGGCTGTGCTCATCCT-3'
Cebpa	5'-CAAGAACAGCAACGAGTACCG-3'	5'-GTCACTGGTCAACTCCAGCAC-3'
Adipoq	5'-AAAGGAGAGCCTGGAGAA-3'	5'-GAATGGGTACATTGGGAACA-3'
Lpl	5'-GGGAGTTTGGCTCCAGAGTTT-3'	5'-TGTGTCTTCAGGGGTCCTTAG-3'
Acta2	5'-GTCCCAGACATCAGGGAGTAA-3'	5'-TCGGATACTTCAGCGTCAGGA-3'
Tagln	5'-CAACAAGGGTCCATCCTACGG-3'	5'-ATCTGGGCGGCCTACATCA-3'
Myl9	5'-ACAGCGCCGAGGACTTTTC-3'	5'-AGACATTGGACGTAGCCCTCT-3'
Tgfb1	5'-CTCCCGTGGCTTCTAGTGC-3'	5'-GCCTTAGTTTGGACAGGATCTG-3'
Col1a1	5'-GCTCCTCTTAGGGGCCACT-3'	5'-CCACGTCTCACCATTGGGG-3'
Col9a1	5'-AGGGGGACCCTTAATGCCT-3'	5'-AGGGGGACCCTTAATGCCT-3'
Col9a3	5'-GGAATGCCGGGGTTCAAGG-3'	5'-AGTCCTCTTAATCCTCGTGGG-3'
Col10a1	5'-TTCTGCTGCTAATGTTCTTGACC-3'	5'-GGGATGAAGTATTGTGTCTTGGG-3'
Col11a2	5'-GAAGGGTGCTCGTGGGAAA-3'	5'-GAGGGCCTGGGTATCCTAGAG-3'
Csf1	5'-ATGAGCAGGAGTATTGCCAAGG-3'	5'-TCCATTCCCAATCATGTGGCTA-3'
Cxcl12	5'-CTGTGCCCTTCAGATTGT-3'	5'-AGCTTTCTCCAGGTACTCTT-3'
Kitl	5'-TCCGAAGAGGCCAGAAA-3'	5'-TGTCAGATGCCACCATAAAG-3'
Vegfa	5'-GCACATAGAGAGAATGAGCTTCC-3'	5'-CTCCGCTCTGAACAAGGCT-3'
Vegfc	5'-GAGGTCAAGGCTTTTGAAGGC-3'	5'-CTGTCCTGGTATTGAGGGTGG-3'
Il7	5'-TTCCTCCACTGATCCTTGTTCT-3'	5'-AGCAGCTTCCTTTGTATCATCAC-3'
Actb	5'-GGCTGTATTCCCCTCCATCG-3'	5'-CCAGTTGGTAACAATGCCATGT-3'
